# Halogenated butyrolactones from the biomass-derived synthon levoglucosenone

**DOI:** 10.3762/bjoc.21.175

**Published:** 2025-10-29

**Authors:** Johannes Puschnig, Martyn Jevric, Ben W Greatrex

**Affiliations:** 1 Faculty of Medicine and Health, University of New England, Armidale, NSW, 2351, Australia

**Keywords:** butyrolactone, cyrene, fluorine, halogenation, levoglucosenone

## Abstract

Halogenated butyrolactones are found in a variety of bioactive materials and used for the construction of nucleoside analogues. Short procedures for their synthesis have been developed starting with levoglucosenone, which can be obtained in a single step from the pyrolysis of acid-treated cellulose. The processes use inexpensive reagents for the stereoselective C3 functionalization of the bicyclic ring system, with a subsequent Baeyer–Villiger oxidation affording the fluorinated, chlorinated, and brominated dideoxyribonolactones.

## Introduction

**Figure 1:**
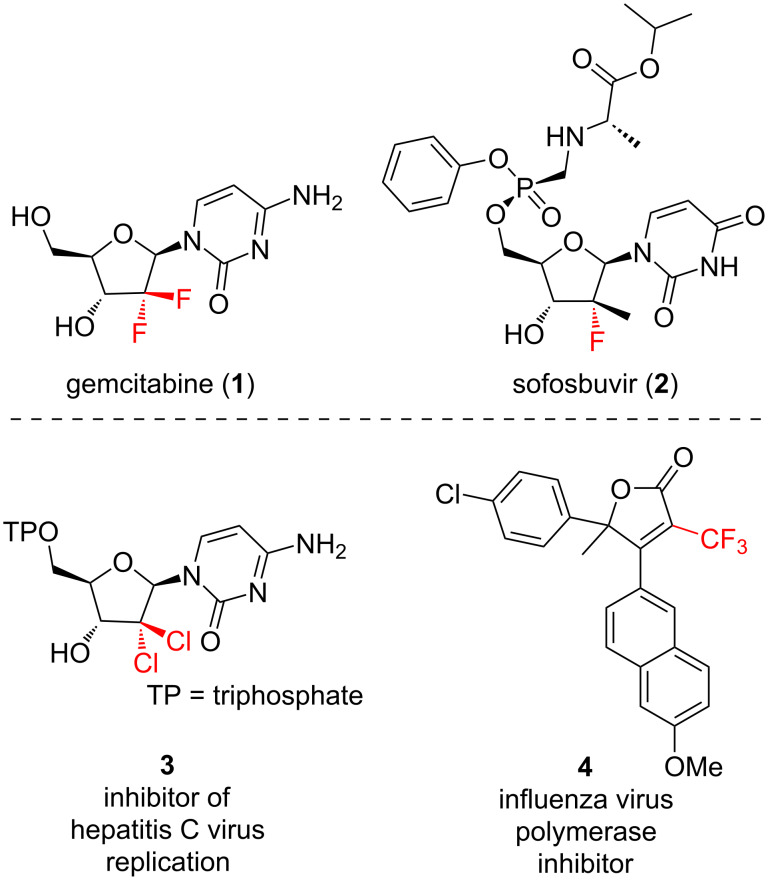
Halogen-containing butyrolactone-derived bioactives.

The γ-butyrolactone ring is a privileged scaffold found in natural products and can be used as a valuable intermediate in synthesis [1–2]. Several nucleoside analogue drugs are prepared using γ-butyrolactones, that when reduced give pentose sugars that can be used as glycosyl donors [3–4]. A number of these clinically used drugs contain fluorine as a hydroxy bioisostere at C2, most notably gemcitabine (**1**) and sofosbuvir (**2**). Fluorination at C2 in the nucleoside results in metabolic stability and resistance to hydrolysis as it destabilizes the formation of a C1 oxocarbenium ion [5–6]. Trifluoromethylated γ-butyrolactones also find applications as antiviral agents, for example, lactone **4** which has activity against influenza [7], while chlorinated analogues such as **3** have demonstrated activity against hepatitis C (Figure 1) [8]. Stereoselective methods to access halogenated γ-butyrolactones are therefore valuable, as they enable access to nucleoside analogues which have applications in treating cancer and certain infections.

The preparation of 2-halo-2-deoxy-ᴅ-ribose derivatives can be achieved via the modification of the parent sugar [9–10] or chain-elongation strategies from lower homologues. For example, Castro and co-workers have demonstrated the synthesis of a dichlorinated 2-deoxypentose via the addition of dichloro-magnesium enolates to protected ᴅ-glyceraldehyde [11]. In 1988, Hertel et al. from the Lilly laboratories published the first synthesis of the clinically important anticancer agent gemcitabine, using an intermediate γ-butyrolactone constructed using protected ᴅ-glyceraldehyde and ethyl bromodifluoroacetate under Reformatsky conditions [12]. While this reaction is still used in recent patents concerning the synthesis of gemcitabine, the drug’s commercial success has driven interest in alternative procedures for its synthesis [13], as well as the extensive evaluation of other halogenated derivatives [14–15].

**Scheme 1:**
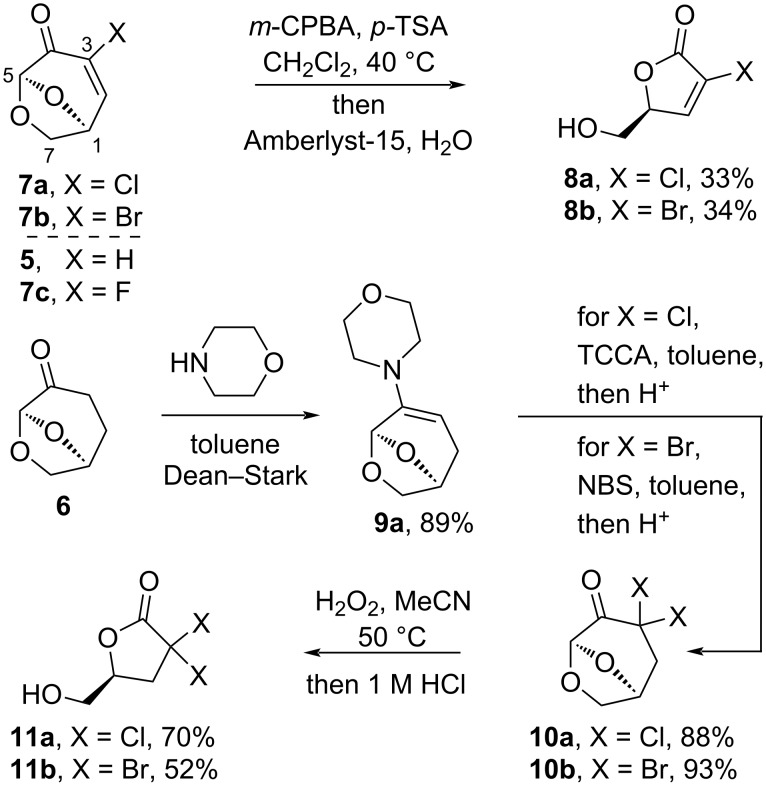
Preparation of chlorinated and brominated lactones **8a**,**b** and **11a**,**b**.

In recent years, the chiral biomass derivatives levoglucosenone (LGO, **5**) and its reduced form Cyrene^®^ (**6**) have gained increased attention as platforms for drug discovery [16–19]. The bicyclic ketone **5** is the major product from the pyrolysis of acid-treated cellulose [20], while its reduced form **6**, which is sold as a solvent, is an inexpensive commercially available reagent (Scheme 1) [21]. Monohalogenation of LGO giving chloride **7a** [22] and bromide **7b** [23] is readily achieved in a single step, however, fluorinated **7c**, which is a potent inflammasone inhibitor (0.8 ± 0.5 µM), has only been reported in a 12-step procedure starting with glucose in an overall yield of 2% [24]. Enol esters [25], enolates [26], enamines [27–28], and silyl enol ethers [29], some of which can be derived from LGO, have been used in electrophilic fluorination and trifluoromethylation strategies. It was envisaged that halogenation could be combined with the Baeyer–Villiger oxidation which yields the butyrolactones by excision of C5, a reaction which is tolerant to substitution at C3 and can be carried out on a kilogram scale [30]. The present work was focussed on the development of additional halogenation reactions for **5** to give substrates for the Baeyer–Villiger oxidation resulting in halogenated butyrolactones, which is an unexplored chemical space for this biomass derivative.

## Results and Discussion

The halogenated LGO derivatives **7a** and **7b** were prepared using literature procedures [22–23]. The reaction of alkenyl halides **7a** and **7b** with the green oxidant H_2_O_2_ gave trace conversion after 3 days; however, the reaction using *m-*CPBA catalyzed with *p-*TSA was complete in 24 hours and afforded the halogenated lactones **8a** and **8b** in 33% and 34% yield, respectively (Scheme 1). We have recently reported the C3 monochlorination and bromination of ketone **6** via enamine **9a** [31], and it was envisaged that **9a** would be a suitable substrate to achieve the double halogenation using an excess of electrophilic halogen. When enamine **9a** was treated with 1.0 mol equivalent of trichloroisocyanouric acid (TCCA), a reagent which can transfer all three chlorine atoms [32], dichlorinated ketone **10a** was obtained in 88% yield following acidic workup. Similarly, treatment of enamine **9a** with 3.0 equivalents of *N-*bromosuccinimide and acidic hydrolysis gave dibrominated material **10b** in excellent yield. This procedure is attractive due to the ready availability of enamine **9a**; however, direct double halogenation of **6** may also be possible using an excess of halogenation agent in DMSO [31]. The Baeyer–Villiger oxidation using H_2_O_2_ in MeCN gave the desired chiral lactones **11a** and **11b** in moderate to good yield following an acidic workup to cleave the intermediate formate ester.

**Scheme 2:**
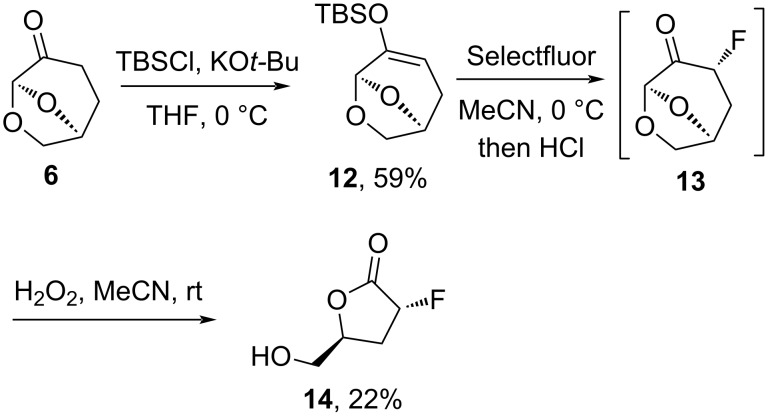
Preparation of fluorinated lactone **14**.

Fluorination of enamine **9a** with Selectfluor (SF) resulted only in hydrolysis with conditions adapted from Peng and Shreeves work [28]; and likewise, the base-promoted (KO*t-*Bu, LHMDS) fluorination of ketone **6** with Selectfluor was unsuccessful. However, when ketone **6** was converted into the silyl enol ether **12** and treated with Selectfluor, fluorinated ketone **13** was formed as the major product after aqueous workup (Scheme 2). When the solution of **13** was concentrated following flash column chromatography, rapid decomposition was observed, which may explain failures with many fluorination attempts. Maintaining some residual solvent allowed for the characterization of compound **13** by NMR spectroscopy. Stereochemical assignment in **13** was based on a large trans-axial coupling constant *J*_H2_*_exo_*_-H3_ = 10.6 Hz in the ^1^H NMR spectrum and interactions between H3*_endo_* and H7*_endo_* on the oxymethylene bridge in the 2D NOESY NMR spectrum. Given the instability of fluoro-ketone **13**, a one-pot procedure was developed in which the reaction mixture containing **13** was subjected to the Baeyer–Villiger oxidation with H_2_O_2_ giving the fluorinated and stable lactone **14**, which is the expected kinetic product in 22% yield.

**Scheme 3:**
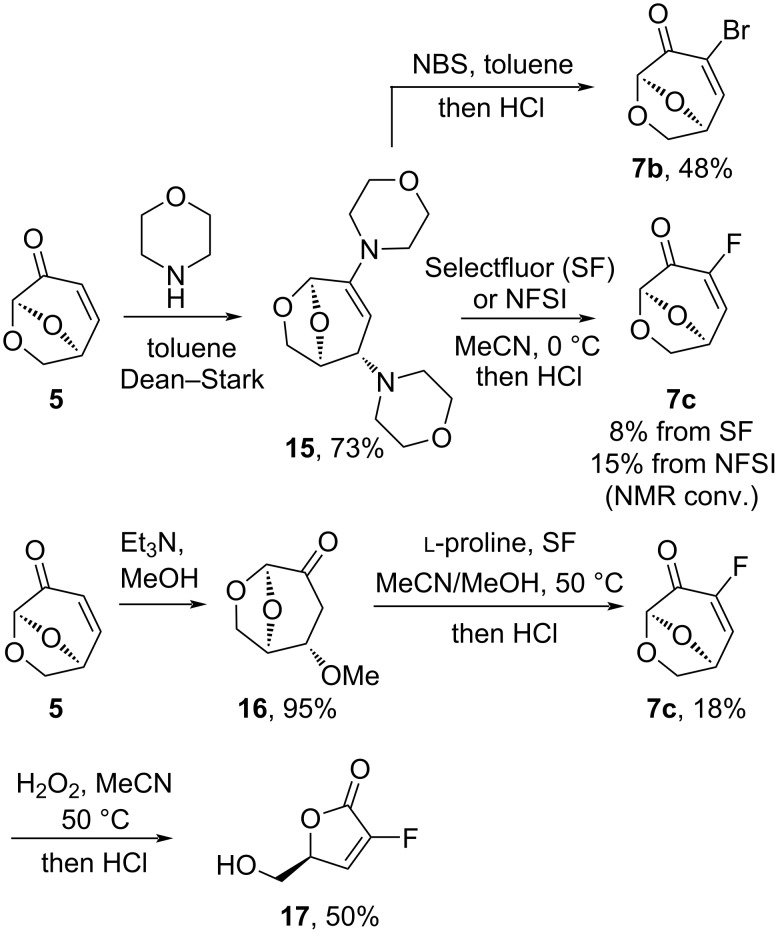
Fluorination of LGO (**5**) and conversion to lactone **17**.

Fluorination of α,β-unsaturated ketones is a more challenging transformation than the halogenation reactions presented in Scheme 1 [33]. The previous reports of enamines as suitable substrates led us to prepare and examine enamine **15**, which was generated from **5** with 3.0 equivalents of morpholine via an aza-Michael addition and condensation (Scheme 3). It was envisaged that the β-amino group could act as a stable functional group resulting in Baylis–Hillman-like reactivity. An extensive survey of reaction conditions using Selectfluor and *N*-fluorobenzenesulfonimide (NFSI) as the sources of electrophilic fluorine in the best case gave 15% conversion for NFSI and less than 8% isolated yield of **7c** for SF. The major product in these reactions was **5**, due to a β-elimination of the amine. In comparison, the reaction of **15** with NBS afforded **7b** in an unoptimized yield of 48% after hydrolysis, which demonstrated that halogenation reactions with **15** were viable, but dependent on the characteristics of the electrophile. To avoid the elimination of the amine, β-methyl ether **16** was prepared. The reaction of ketone **16** with Selectfluor promoted by ʟ-proline in MeOH/MeCN, which ensured that the equilibrium between was biased to **16** and not **5**, gave full consumption of the starting material in 2 hours and the fluorinated product **7c** was obtained in 18% yield. Attempts to combine the methanol addition reaction and the fluorination starting with LGO proceeding through **16** were unsuccessful. Although the yield was low, the synthesis of **7c** via this method represents a substantial improvement on previous approaches to this material [24]. The Baeyer–Villiger oxidation of **7c** was uneventful and the fluorinated alkene **17** was isolated in 50% yield.

**Scheme 4:**
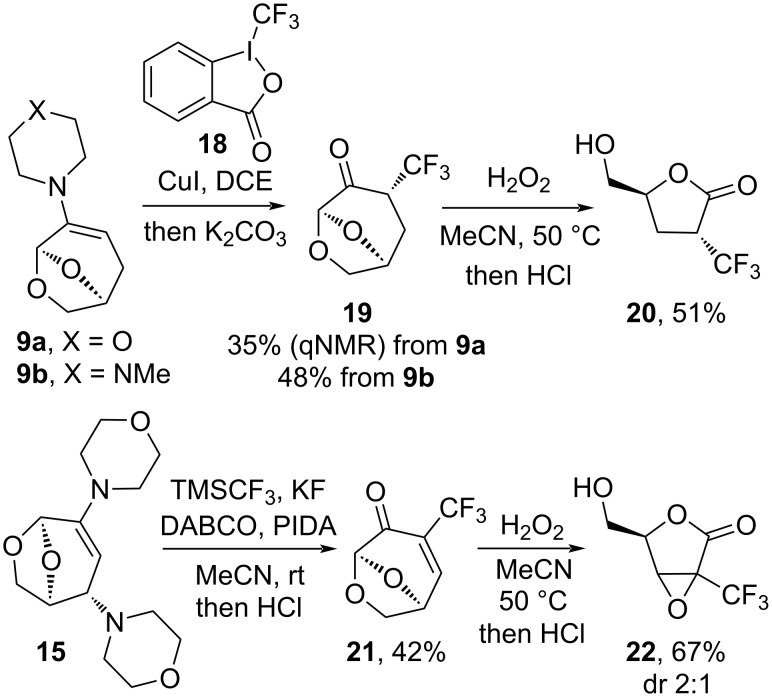
Trifluoromethylation of **9a**,**b** and **15** and subsequent Baeyer–Villiger oxidation.

The α-trifluoromethylation of ketones and aldehydes can be performed using CF_3_ donors in copper-catalyzed [34], base- and Lewis acid-mediated reactions [35–37]. The reaction of enamine **9a** with Togni’s reagent (**18**) and subsequent hydrolysis gave the substituted derivative **19** in 35% yield (qNMR) (Scheme 4). The yield was improved using the *N*-methylpiperazine-derived enamine **9b**, which was completely consumed in 6 hours at room temperature. Following acidic hydrolysis of the enamine, a mixture of α-trifluoromethylated ketone diastereomers was obtained, and subsequent epimerization with K_2_CO_3_ afforded **19** as a single stereoisomer in 48% yield. The equatorial position of the trifluoromethyl group was established based on NOE interactions between H3*_endo_* and H7*_endo_* in the oxymethylene bridge. Subsequent Baeyer–Villiger oxidation of **19** gave lactone **20** in moderate yield. Attempts to improve the yield of **19** using base-promoted trifluoromethylation [38] of **6** gave only traces of the desired product.

Methods for the installation of a CF_3_-group on enones are limited, although approaches have been applied to quinones, uracils, flavones or arylenones via radical pathways [39–42]. As per the fluorination reactions, we envisaged that a leaving group in the β-position could mask the double bond when incorporating the trifluoromethyl group into **5**. When enamine **15** was subjected to the same conditions established for the trifluoromethylation of enamine **9b**, only trace amounts of the desired product were detected. It was found that generating the electrophilic CF_3_ species in situ was crucial for this transformation. By modifying a procedure for the trifluoromethylation of ketene dithioacetals reported by Liu and co-workers using TMSCF_3_ [43], rapid consumption of enamine **15** was observed. Acidic treatment of the intermediate promoted the hydrolysis/elimination cascade to give enone **21** in 42% yield. Baeyer–Villiger oxidation of this highly activated alkene promoted an epoxidation/Baeyer–Villiger oxidation cascade to yield lactone **22** in 67% yield (dr 2:1). The low diastereoselectivity suggested that the epoxidation happened subsequent to the ring contraction, as epoxidations of **5** are highly stereoselective [44]. Unambiguous assignment of configuration for the diastereomers was not possible on the basis of the selective 1D NOE spectra due to the lack of informative crosspeaks. Limiting the amount of oxidant (H_2_O_2_ or peracetic acid) resulted in mixtures, suggesting that the Baeyer–Villiger reaction without epoxidation may be challenging.

## Conclusion

In conclusion, we have successfully established halogenation strategies of the biomass derivates **5** and **6**, including fluorinations and trifluoromethylation. Baeyer–Villiger oxidations of these materials provide access to chiral halogenated lactones, which could be useful in the enantioselective synthesis of valuable drug precursors. The syntheses feature the use of readily available and cheap starting materials, and we have also demonstrated some of these transformations on a gram scale.

## Supporting Information

File 1 Experimental section, characterization data and copies of spectra.

## Data Availability

Data generated and analyzed during this study is openly available in Zenodo.org at https://doi.org/10.5281/zenodo.15803415.

## References

[1] Seitz M, Reiser O (2005). Curr Opin Chem Biol.

[2] Hur J, Jang J, Sim J (2021). Int J Mol Sci.

[3] Hoffmann H M R, Rabe J (1985). Angew Chem, Int Ed Engl.

[4] Okabe M, Sun R C, Tam S Y K, Todaro L J, Coffen D L (1988). J Org Chem.

[5] Marquez V E, Tseng C K H, Mitsuya H, Aoki S, Kelley J A, Ford H, Roth J S, Broder S, Johns D G, Driscoll J S (1990). J Med Chem.

[6] Pankiewicz K W (2000). Carbohydr Res.

[7] Mizuta S, Makau J N, Kitagawa A, Kitamura K, Otaki H, Nishi K, Watanabe K (2018). ChemMedChem.

[8] Pinho P, Kalayanov G, Westerlind H, Rosenquist Å, Wähling H, Sund C, Almeida M, Ayesa S, Tejbrant J, Targett-Adams P (2017). Bioorg Med Chem Lett.

[9] Mikhailopulo I A, Sivets G G (1996). Synlett.

[10] Larsen C H, Ridgway B H, Shaw J T, Smith D M, Woerpel K A (2005). J Am Chem Soc.

[11] Rague B, Chapleur Y, Castro B (1982). J Chem Soc, Perkin Trans 1.

[12] Hertel L W, Kroin J S, Misner J W, Tustin J M (1988). J Org Chem.

[13] Brown K, Dixey M, Weymouth-Wilson A, Linclau B (2014). Carbohydr Res.

[14] Cini E, Barreca G, Carcone L, Manetti F, Rasparini M, Taddei M (2018). Eur J Org Chem.

[15] Zhou S, Mahmoud S, Liu P, Zhou L, Ehteshami M, Bassit L, Tao S, Domaoal R A, Sari O, Schutter C D (2017). J Med Chem.

[16] Camp J E, Greatrex B W (2022). Front Chem (Lausanne, Switz).

[17] Comba M B, Tsai Y-h, Sarotti A M, Mangione M I, Suárez A G, Spanevello R A (2018). Eur J Org Chem.

[18] M. Sarotti A, M. Zanardi M, A. Spanevello R (2012). Curr Org Synth.

[19] Stanfield M K, Terry R S, Smith J A, Thickett S C (2023). Polym Chem.

[20] Halpern Y, Riffer R, Broido A (1973). J Org Chem.

[21] Marathianos A, Liarou E, Hancox E, Grace J L, Lester D W, Haddleton D M (2020). Green Chem.

[22] Sarotti A M, Spanevello R A, Suárez A G (2011). Tetrahedron Lett.

[23] Ward D D, Shafizadeh F (1981). Carbohydr Res.

[24] Goto K, Ideo H, Tsuchida A, Hirose Y, Maruyama I, Noma S, Shirai T, Amano J, Mizuno M, Matsuda A (2018). Bioorg Med Chem.

[25] Wood S H, Etridge S, Kennedy A R, Percy J M, Nelson D J (2019). Chem – Eur J.

[26] Davis F A, Zhou P, Murphy C K, Sundarababu G, Qi H, Han W, Przeslawski R M, Chen B-C, Carroll P J (1998). J Org Chem.

[27] Timofeeva D S, Ofial A R, Mayr H (2018). J Am Chem Soc.

[28] Peng W, Shreeve J M (2005). J Org Chem.

[29] Fujisawa H, Takeuchi Y (2002). J Fluorine Chem.

[30] Bonneau G, Peru A A M, Flourat A L, Allais F (2018). Green Chem.

[31] Puschnig J, Sumby C J, Greatrex B W (2025). Eur J Org Chem.

[32] Gaspa S, Carraro M, Pisano L, Porcheddu A, De Luca L (2019). Eur J Org Chem.

[33] Champagne P A, Desroches J, Hamel J-D, Vandamme M, Paquin J-F (2015). Chem Rev.

[34] Li L, Chen Q-Y, Guo Y (2014). J Org Chem.

[35] Iseki K, Nagai T, Kobayashi Y (1993). Tetrahedron Lett.

[36] Charpentier J, Früh N, Togni A (2015). Chem Rev.

[37] Allen A E, MacMillan D W C (2010). J Am Chem Soc.

[38] Matoušek V, Togni A, Bizet V, Cahard D (2011). Org Lett.

[39] Nagib D A, MacMillan D W C (2011). Nature.

[40] Fang Z, Ning Y, Mi P, Liao P, Bi X (2014). Org Lett.

[41] Wang X, Ye Y, Ji G, Xu Y, Zhang S, Feng J, Zhang Y, Wang J (2013). Org Lett.

[42] Ilchenko N O, Janson P G, Szabó K J (2013). Chem Commun.

[43] Xu C, Liu J, Ming W, Liu Y, Liu J, Wang M, Liu Q (2013). Chem – Eur J.

[44] Ledingham E T, Greatrex B W (2018). Tetrahedron.

