# Supplementary material for: Halogenated butyrolactones from the biomass-derived synthon levoglucosenone
Source: Beilstein J Org Chem. 2025 Oct 29;21:2297–301. doi: 10.3762/bjoc.21.175 (PMC12581009; doi:10.3762/bjoc.21.175)

BEILSTEIN JOURNAL OF ORGANIC CHEMISTRY

Supporting Information

for

Halogenated butyrolactones from the biomass-derived synthon levoglucosenone

Johannes Puschnig, Martyn Jevric and Ben W. Greatrex

Beilstein J. Org. Chem. **2025,** *21,* 2297–2301. doi:10.3762/bjoc.21.175

Experimental section, characterization data and copies of spectra

License and Terms: This is a supporting information file under the terms of the Creative Commons Attribution License (https://creativecommons.org/licenses/by/4.0). Please note that the reuse, redistribution and reproduction in particular requires that the author(s) and source are credited and that individual graphics may be subject to special legal provisions.
The license is subject to the *Beilstein Journal of Organic Chemistry* terms and conditions: (https://www.beilstein-journals.org/bjoc/terms)

General experimental

Chemical reagents were commercially available and were used as purchased. Except as indicated, reactions were magnetically stirred and monitored by NMR spectroscopy, GC–MS or thin-layer chromatography (TLC) using silica plates (silica gel 60 F254). Flash chromatography was performed on silica gel 60, using a moderate pressure applied via compressed air, and solvent mixtures are given as v/v. Solvents were removed using a rotary evaporator with a bath temperature of 40 °C and pressure between 10 and 700 mbar. ^{1}H NMR was performed on a 500 MHz Bruker AVANCE III spectrometer at 298 K, and the spectra were referenced to residual solvent $CDCl_3$ (δ 7.26 ppm). ^{13}C NMR spectra were recorded at 125 MHz, and referenced to the residual solvent ($CDCl_3$, δ 77.0 ppm). ^{19}F NMR spectra were recorded at 471 MHz, and referenced to the internal standard hexafluorobenzene (−164.90 ppm). Reactions were followed by gas chromatography with (GC)–MS analyses performed using an Agilent Technologies 7890A GC-System coupled with an Agilent 5975C mass-selective detector (triple-axis detector) using an HP-5MS Agilent column (30 m × 250 µm). Operating conditions were as follows: Injector: split ratio 10:1; inlet temperature: 250 °C; carrier gas: helium, 1.0 mL/min, constant flow; column temperature 50˚C (5 minute hold) heated at 20 °C per minute to 250°C (5 minute hold). MS was acquired at −70 eV using a mass scan range of *m*/*z* 30–700. Optical rotation was measured on a Rudolph Research Analytical Autopol 1 polarimeter operating at the sodium D line. Melting points were measured using differential scanning calorimetry. ESI–MS were recorded using an Agilent 6120 Quadrupole detector and EI–MS were recorded using a 5975C detector. High-resolution electrospray mass spectra were acquired on a Waters Xevo G2X2 QToF electrospray mass spectrometer at Callaghan Innovation, Wellington, New Zealand. Compounds **7a** [1], **9a** [2], **16** [3], and trifluoromethylating agent **18** [4] were prepared according to literature procedures.

Preparation of compounds

Synthesis of (1*S*,5*R*)-3-Bromo-6,8-dioxabicyclo[3.2.1]oct-2-en-4-one (7b) using 15. To a stirred solution of **15** (1.50, 5.32 mmol) in toluene (20 mL) was added *N*-bromosuccinimide (994 mg, 5.38 mmol), and the resultant mixture stirred in the dark at rt for 30 min. To the mixture was added petroleum ether (20 mL) and the resulting suspension filtered through Celite then the precipitate was washed with toluene (20 mL). The filtrate was concentrated under reduced pressure and the residue suspended in 2 M HCl and stirred for 10 min at rt. The aqueous layer was extracted with CH_2Cl_2 (2 × 50 mL), the combined organic layers were dried (Na_2SO_4) and the solvent removed under reduced pressure to afford **7b** as an orange solid (518 mg, 48%). ^{1}H NMR (500 MHz, $CDCl_3$) δ 7.62 (app. d, *J* = 5.1 Hz, 1H), 5.55 (d, *J* = 2.3 Hz, 1H), 5.04 (ddd, *J* = 5.1, 4.6, 0.5 Hz, 1H), 3.89 (ddd, *J* = 7.0, 4.6, 1.5 Hz, 1H), 3.83 (dd, *J* = 7.0, 1.0 Hz, 1H) ; ^{13}C NMR (125 MHz, $CDCl_3$) δ 182.3, 147.6, 122.5, 100.9, 73.5, 66.6. Spectroscopic data was in accordance with literature [5].

(1*S*,5*R*)-3-Fluoro-6,8-dioxabicyclo[3.2.1]oct-2-en-4-one (7c). To a solution of methoxy ether **16** (500 mg, 3.2 mmol) in MeOH (6 mL) and MeCN (6 mL) was added ʟ-proline (727 mg, 6.3 mmol) and Selectfluor (1.35 g, 3.8 mmol), and the mixture heated to 50 °C for 2 h. A 2 M HCl (1 mL) solution was then added and after 5 min the mixture was concentrated under reduced pressure and the residue purified by flash column chromatography (petroleum ether/EtOAc 3:1) to yield **7c** as a colourless oil (80 mg, 18%). R_f 0.5 (petroleum ether/EtOAc, 3:1); ^{1}H NMR (500 MHz, $CDCl_3$) δ 6.72 (dd, *J* = 10.2, 5.3 Hz, 1H), 5.48 (d, *J* = 7.4 Hz, 1H), 5.14 (ddd, *J* = 10.2, 4.4, 0.4 Hz, 1H), 3.94 (ddd, *J* = 6.9, 4.4, 1.5 Hz, 1H), 3.84 (d, *J* = 6.9, 0.4 Hz, 1H); ^{13}C NMR (125 MHz, $CDCl_3$) δ 182.7 (d, *J* = 17.8 Hz), 152.8 (d, *J* = 280.2 Hz), 123.0 (d, *J* = 8.2 Hz), 101.2 (d, *J* = 6.5 Hz), 71.8 (d, *J* = 7.3 Hz), 67.1 (d, *J* = 2.9 Hz); ^{19}F NMR (471 MHz, $CDCl_3$) δ −134.6. Spectroscopic data was in accordance with literature [6].

(*S*)-3-Chloro-5-(hydroxymethyl)furan-2(5*H*)-one (8a). To a stirred a solution of **7a** (241 mg, 1.5 mmol) in CH_2Cl_2 (6 mL) was added *m*-CPBA (811 mg, 3.3 mmol) and *p*-TSA (259 mg, 1.5 mmol), and the mixture heated to reflux for 24 h. The reaction was quenched by the addition of MnO_2 (200 mg), then Amberlyst 15 (100 mg) and H_2O (0.200 mL) were added and the mixture stirred for 1 h at rt. K_2CO_3 (200 mg) was added, and the mixture was filtered through a pad of Celite and the filtrate concentrated under reduced pressure. The residue was purified by flash column chromatography (petroleum ether/EtOAc 1:1) to yield **8a** as a colourless oil (74 mg, 33%). ^{1}H NMR (500 MHz, $CDCl_3$) δ 7.37 (br s, 1H), 5.16–5.11 (m, 1H), 4.00 (dd, *J* = 12.4, 3.3 Hz, 1H), 3.82 (dd, *J* = 12.4, 4.6 Hz, 1H), 3.15 (br s, 1H); ^{13}C NMR (125 MHz, $CDCl_3$) δ 168.1, 145.4, 125.8, 81.7, 62.0. Spectroscopic data was in accordance with literature [7].

(*S*)-3-Bromo-5-(hydroxymethyl)furan-2(5*H*)-one (8b). To a stirred a solution of **7b** (410 mg, 2.0 mmol) in CH_2Cl_2 (8 mL) was added *m*-CPBA (1.08, 4.4 mmol) and *p*-TSA (344 mg, 2.0 mmol), and the mixture heated to reflux for 24 h. The reaction was quenched by the addition of MnO_2 (200 mg), followed by the addition of Amberlyst 15 (100 mg) and H_2O (0.200 mL) and stirred for 1 h at rt. K_2CO_3 (200 mg) was added, and the mixture filtered through a pad of Celite and then concentrated under reduced pressure. The residue was purified by flash column chromatography (petroleum ether/EtOAc 1:1) to yield **8b** as a colourless solid (131 mg, 34%). ^{1}H NMR (500 MHz, $CDCl_3$) δ 7.52 (d, *J* = 1.7 Hz, 1H), 5.08 (ddd, *J* = 4.9, 3.9, 1.7 Hz, 1H), 4.00 (dd, *J* = 12.3, 3.9 Hz, 1H), 3.84 (dd, *J* = 12.3, 4.9 Hz, 1H); ^{13}C NMR (125 MHz, $CDCl_3$) δ 168.1, 149.6, 114.5, 82.9, 62.3. Spectroscopic data was in accordance with literature [8].

(1*S*,5*R*)-3,3-Dichloro-6,8-dioxabicyclo[3.2.1]octan-4-one (10a). To a solution of enamine **9a** (2.00 g, 10.1 mmol) in toluene (40 mL) was added trichloroisocyanuric acid (2.36 g, 10.1 mmol) and the mixture stirred in the dark at rt for 90 min. The reaction was quenched by the addition of satd. NH_4Cl solution (50 mL), the mixture filtered through a pad of Celite, and the Celite pad washed with EtOAc (50 mL). The filtrate was washed with 2 M HCl (50 mL) and then the aqueous layer extracted with EtOAc (2 × 50 mL). The combined organic layers were dried (Na_2SO_4) then concentrated under reduced pressure. The residue was dissolved in a small amount of CH_2Cl_2, filtered through a small pad of SiO_2, which was washed with CH_2Cl_2 (100 mL), and the filtrate concentrated under reduced pressure to yield **10a** as a colourless oil (1.75 g, 88%). $[\alpha]_D^{22}$ −66 (*c* 1.0, CH_2Cl_2); IR $\tilde{\nu}_{max}$ 2971, 1762, 1734, 1426, 1324 cm^{-1}; ^{1}H NMR (500 MHz, $CDCl_3$) δ 5.49 (s, 1H), 4.76 (dddd, *J* = 5.3, 4.3, 1.9, 0.8 Hz, 1H), 4.44 (dd, *J* = 7.6, 0.7 Hz, 1H), 3.89 (dd, *J* = 7.6, 5.3 Hz, 1H), 3.26–3.16 (m, 2H); ^{13}C NMR (125 MHz, $CDCl_3$) δ 186.2, 100.9, 79.6, 72.9, 66.2, 51.9; HRMS (ESI-TOF) m/z: $[M+H]^+$ calcd for $C_6H_7Cl_2O_3^+$: 196.9767 ; found: 196.9768.

(1*S*,5*R*)-3,3-Dibromo-6,8-dioxabicyclo[3.2.1]octan-4-one (10b). To a solution of enamine **9a** (1.00 g, 5.1 mmol) in toluene (15 mL) was added *N*-bromosuccinimide (2.70 g, 15.3 mmol) and the mixture was stirred in the dark at rt for 6 h. The resulting mixture was washed with 2 M HCl (30 mL), and then the aqueous layer was extracted with CH_2Cl_2 (3 × 50 mL). The combined organic layers were dried (Na_2SO_4), concentrated under reduced pressure, and the residue was purified by flash column chromatography (petroleum ether/EtOAc 4:1) to yield **10b** as a colourless solid (1.35 g, 93%). mp 88–91 °C; $[\alpha]_D^{28}$ −31 (*c* 1.0, CH_2Cl_2); R_f 0.4 (petroleum ether/EtOAc, 4:1); IR $\tilde{\nu}_{max}$ 2974, 2912, 1759, 1748, 1707 cm^{-1}; ^{1}H NMR (500 MHz, $CDCl_3$) δ 5.49 (s, 1H), 4.68–4.64 (m, 1H), 4.52 (d, *J* = 7.6 Hz, 1H), 3.84 (ddd, *J* = 7.6, 5.3, 0.6 Hz, 1H), 3.66 (ddd, *J* = 16.0, 4.9, 0.8 Hz, 1H), 3.39 (dd, *J* = 16.0, 0.6 Hz, 1H); ^{13}C NMR (125 MHz, $CDCl_3$) δ 187.1, 100.4, 73.5, 65.8, 53.9, 53.7; HRMS (ESI-TOF) m/z: $[M+H]^+$ calcd for $C_6H_7Br_2O_3^+$: 284.8756; found: 284.8762.

(*S*)-3,3-Dichloro-5-(hydroxymethyl)dihydrofuran-2(3*H*)-one (11a). To a solution of **10a** (500 mg, 2.5 mmol) in MeCN (10 mL) was added 30% H_2O_2 (2.07 mL, 20.3 mmol) and the mixture heated to 50 °C for 16 h. The reaction was quenched by the addition of Pt/C (5 mg) until the evolution of oxygen had ceased, and then 2 M HCl (1 mL) was added and the resulting mixture stirred at rt for 1 h. The mixture was filtered through a pad of Celite and the volatiles removed under reduced pressure. The residue was purified by flash column chromatography (petroleum ether/EtOAc 1:1) to yield **11a** as a colourless oil (326 mg, 70%). $[\alpha]_D^{28}$ +28 (*c* 1.0, CH_2Cl_2); R_f 0.5 (petroleum ether/EtOAc, 1:1); IR $\tilde{\nu}_{max}$ 3404, 1784, 1437, 1368, 1185 cm^{-1}; ^1H NMR (500 MHz, $CDCl_3$) δ 4.74 (dddd, *J* = 9.0, 6.4, 3.5, 2.6 Hz, 1H), 4.08 (dd, *J* = 13.0, 2.6 Hz, 1H), 3.72 (dd, *J* = 13.0, 3.5 Hz, 1H), 3.10–2.99 (m, 2H), 2.01 (br s, 1H); ^{13}C NMR (125 MHz, $CDCl_3$) δ 167.7, 77.9, 77.5, 61.5, 44.9; HRMS (ESI-TOF) m/z: $[M+H]^+$ calcd for $C_5H_7Cl_2O_3^+$: 184.9767; found: 184.9777.

(*S*)-3,3-Dibromo-5-(hydroxymethyl)dihydrofuran-2(3*H*)-one (11b). To a solution of **10b** (400 mg, 1.4 mmol) in MeCN (6 mL) was added 30% H_2O_2 (1.14 mL, 11.2 mmol) and the mixture heated to 50 °C for 8 h. The reaction was quenched by the addition of Pt/C (5 mg) until the evolution of oxygen had ceased, and then 2 M HCl (1 mL) was added and the resulting mixture stirred at rt for 1 h. The mixture was filtered through a pad of Celite, the filtrate concentrated under reduced pressure and the residue was purified by flash column chromatography (petroleum ether/EtOAc 3:1 to 1:1) to yield **11b** as a colourless oil (199 mg, 52%). $[\alpha]_D^{28}$ +19 (*c* 1.0, CH_2Cl_2); R_f 0.3 (petroleum ether/EtOAc, 1:1); IR $\tilde{\nu}_{max}$ 3412, 1776, 1436, 1343, 1179 cm^{-1}; ^1H NMR (500 MHz, $CDCl_3$) δ 4.74 (dddd, *J* = 9.6, 5.1, 3.7, 2.8 Hz, 1H), 4.07 (ddd, *J* = 13.0, 5.5, 2.8 Hz, 1H), 3.72 (ddd, *J* = 13.0, 7.3, 3.7 Hz, 1H), 3.27 (dd, *J* = 14.5, 9.6 Hz, 1H), 3.17 (dd, *J* = 14.5, 5.1 Hz, 1H), 1.97 (dd, *J* = 7.3, 5.5 Hz, 1H); ^{13}C NMR (125 MHz, $CDCl_3$) δ 168.6, 78.6, 61.4, 47.7, 47.2; HRMS (ESI-TOF) m/z: $[M+H]^+$ calcd for $C_5H_7Br_2O_3^+$: 272.8756; found: 272.8761.

(((1*S*,5*R*)-6,8-Dioxabicyclo[3.2.1]oct-3-en-4-yl)oxy)(*tert*-butyl)dimethylsilane (12). Ketone **6** (2.00 g, 15.6 mmol) was dissolved in THF (60 mL) and cooled in an ice bath to 0 °C and then KO*t*-Bu (3.50 g, 31.2 mmol) and TBDMSCl (3.52 g, 23.4 mmol) were added. After stirring at 0 °C for 90 min, saturated NaCl solution (100 mL) was added and the mixture extracted with EtOAc (2 × 100 mL). The combined organic layers were dried over Na_2SO_4, the volatiles removed under reduced pressure, and the residue purified by flash column chromatography (petroleum ether/EtOAc 95:5) to yield **12** as a yellowish oil (2.23 g, 59%). $[\alpha]_D^{27}$ +56 (*c* 1.0, CH_2Cl_2); IR $\tilde{\nu}_{max}$ 2930, 2857, 1738, 1663, 1214 cm^{-1}; ^1H NMR (500 MHz, $CDCl_3$) δ 5.16 (d, *J* = 1.2 Hz, 1H), 4.64–4.61 (m, 2H), 3.91 (ddd, *J* = 7.1, 6.2, 1.7 Hz, 1H), 3.63 (dd, *J* = 7.1, 1.8 Hz, 1H), 2.71 (dddd, *J* = 16.8, 3.8, 2.4, 1.7 Hz, 1H), 1.83 (ddd, *J* = 16.8, 4.8, 1.0 Hz, 1H), 0.92 (s, 9H), 0.16 (s, 3H), 0.15 (s, 3H); ^{13}C NMR (125 MHz, $CDCl_3$) δ 150.3, 99.1, 96.7, 71.6, 68.1, 30.3, 25.6, 18.0, −4.6, −4.8; HRMS (ESI-TOF) m/z: $[M+H]^+$ calcd for $C_{12}H_{23}O_3Si^+$: 243.1411; found: 243.1415.

(3*R*,5*S*)-3-Fluoro-5-(hydroxymethyl)dihydrofuran-2(3*H*)-one (14). To a solution of silyl enol ether **12** (1.00 g, 4.1 mmol) in MeCN at 0 °C was added Selectfluor (1.60 g, 4.5 mmol) and the mixture stirred for 10 min. The reaction was allowed to warm to rt and stirred for 1 h, and then 2 M HCl (5 mL) was added. To this mixture was added 30% H_2O_2 (2.1 mL, 20.7 mmol) and the resulting solution stirred at rt for 6 h. The reaction mixture was quenched by the addition of Pt/C (5 mg) until the evolution of oxygen had ceased, and then the mixture was filtered through a pad of Celite and concentrated under reduced pressure. The residue was purified by flash column chromatography (petroleum ether/EtOAc 1:3) to yield **14** as a colourless oil (124 mg, 22%). $[\alpha]_D^{27}$ +30 (*c* 0.5, CH_2Cl_2); R_f 0.3 (petroleum ether/EtOAc, 1:3); IR $\tilde{\nu}_{max}$ 3412, 2927, 1768, 1194 cm^{-1}; ^1H NMR (500 MHz, $CDCl_3$) δ 5.38 (ddd, *J* = 52.4, 8.2, 6.9 Hz, 1H), 4.79–4.74 (m, 1H), 3.99 (ddd, *J* = 12.5, 2.3, 1.3 Hz, 1H), 3.64 (dd, *J* = 12.5, 2.3 Hz, 1H), 2.66 (dddd, *J* = 13.8, 13.3, 8.2, 3.3 Hz, 1H), 2.52 (dddd, *J* = 27.1, 13.8, 8.5, 6.9 Hz, 1H), 2.43 (br s, 1H); ^{13}C NMR (125 MHz, $CDCl_3$) δ 172.5 (d, *J* = 20.6 Hz), 85.7 (d, *J* = 186.9 Hz), 78.1 (d, *J* = 4.1 Hz), 63.4, 31.4 (d, *J* = 20.4 Hz); ^{19}F NMR (471 MHz, $CDCl_3$) δ −192.5. HRMS (ESI-TOF) m/z: $[M+H]^+$ calcd for $C_5H_8FO_3^+$: 135.0452; found: 135.0454.

4,4'-((1*S*,2*S*,5*R*)-6,8-Dioxabicyclo[3.2.1]oct-3-ene-2,4-diyl)dimorpholine (15). To a stirred solution of **5** (11.2 mL, 119 mmol) in toluene (200 mL) was added morpholine (30.8 mL, 357 mmol), and the resultant mixture was refluxed under Dean–Stark conditions for 20 h. The mixture was concentrated under reduced pressure and the residue recrystallised from toluene/petroleum ether 9:1 to yield **15** as a yellowish solid (24.5 g, 73%). mp 126–128 °C; $[\alpha]_D^{31}$ −292 (*c* 1.0, CH_2Cl_2); IR $\tilde{\nu}_{max}$ 2853, 1635, 1452, 1262, 1222, 1115, 999, 978 cm^{-1}; ^1H NMR ($CDCl_3$, 500 MHz): δ 5.51 (d, *J* = 1.3 Hz, 1H), 4.82 (app. d, *J* = 6.3, 1H), 4.42–4.39 (m, 1H), 3.90 (dd, *J* = 7.3, 6.3 Hz, 1H), 3.78–3.70 (m, 5H), 3.73–3.64 (m, 3H), 3.47 (dd, *J* = 7.3, 1.9 Hz, 1H), 2.91–2.84 (m, 2H), 2.82–2.64 (m, 7H); ^{13}C NMR ($CDCl_3$, 125 MHz): δ 150.4, 96.9, 93.4, 72.3, 67.5, 66.5, 66.4, 63.3, 50.1, 48.2.

(*S*)-3-Fluoro-5-(hydroxymethyl)furan-2(5*H*)-one (17). To a solution of **7c** (75 mg, 0.52 mmol) in MeCN (2 mL) was added 30% H_2O_2 (0.26 mL, 2.6 mmol) and the mixture heated to 50 °C for 16 h. The reaction was quenched by the addition of Pt/C (5 mg) and after the evolution of oxygen had ceased, 2 M HCl (1 mL) was added. The mixture was filtered through a pad of Celite, the filtrate concentrated under reduced pressure, and then the residue was purified by column chromatography (petroleum ether/EtOAc 1:3) to yield **17** as a colourless oil (34.2 mg, 50%). $[\alpha]_D^{28}$ −42 (*c* 1.0, CH_2Cl_2); R_f 0.4 (petroleum ether/EtOAc, 1:3); IR $\tilde{\nu}_{max}$ 3423, 2923, 1959, 1679 cm^{-1}; ^1H NMR (500 MHz, $CDCl_3$) δ 6.75–6.70 (m, 1H), 5.12–5.05 (m, 1H), 4.00 (dd, *J* = 12.3, 3.3 Hz, 1H), 3.80 (dd, *J* = 12.3, 4.9 Hz, 1H), 2.42 (br. s, 1H); ^{13}C NMR (126 MHz, $CDCl_3$) δ 164.5 (d, *J* = 35.6 Hz), 149.0 (d, *J* = 280.7 Hz), 123.1 (d, *J* = 6.6 Hz),

78.0 (d, J = 6.5 Hz), 62.5 (d, J = 2.8 Hz); ^{19}F NMR (471 MHz, $CDCl_3$) δ −142.3; HRMS (ESI-TOF) m/z: $[M+H]^+$ calcd for $C_5H_6FO_3^+$: 133.0295 ; found: 133.0307.

1-((1*S*,5*R*)-6,8-Dioxabicyclo[3.2.1]oct-3-en-4-yl)-4-methylpiperazine (9b). To a solution of **6** (5.00 g, 39.0 mmol) in toluene (200 mL) was added *N*-methylpiperazine (8.67 mL, 78.0 mmol) and the mixture was heated to reflux under Dean–Stark conditions for 14 h. The mixture was allowed to cool to rt and concentrated under reduced pressure. The crude material was recrystallised from hot Et_2O (100 mL), then dried under reduced pressure to give **9b** as a yellowish solid (6.22 g, 76%). mp 81–84 °C; $[\alpha]_D^{22}$ −20 (*c* 1.0, CH_2Cl_2); IR $\tilde{\nu}_{max}$ 2970, 2944, 2797, 1644, 1455 cm^{-1}; ^{1}H NMR (500 MHz, $CDCl_3$) δ 5.47 (s, 1H), 4.68–4.64 (m, 1H), 4.42–4.37 (m, 1H), 3.92–3.86 (m, 1H), 3.68 (br d, J = 7.0 Hz, 1H), 2.90–2.80 (m, 2H), 2.79–2.70 (m, 3H), 2.50–2.39 (m, 4H), 2.28 (s, 3H), 1.87 (dd, J = 17.0, 4.6 Hz, 1H); ^{13}C NMR (125 MHz, $CDCl_3$) δ 147.4, 97.4, 94.1, 72.1, 68.4, 54.7, 48.1, 46.1, 30.9.

(1*S*,3*R*,5*R*)-3-(Trifluoromethyl)-6,8-dioxabicyclo[3.2.1]octan-4-one (19). To a solution of enamine **9b** (750 mg, 3.6 mmol) in 1,2-dichloroethane (15 mL) was added copper(I)iodide (68 mg, 0.36 mmol) and 1-(trifluoromethyl)-1λ^3-benzo[*d*][1,2]iodaoxol-3(1*H*)-one (2.70 g, 4.3 mmol, 50% on Celite) and the mixture stirred under an N_2 atmosphere at rt for 6 h. The reaction was quenched by the addition of 2 M HCl (25 mL), then extracted with EtOAc (4 × 25 mL). The combined organic layers were dried (Na_2SO_4), the volatiles removed under reduced pressure, and the residue purified by flash column chromatography (petroleum ether/EtOAc 4:1) to yield **19** as a colourless solid (338 mg, 48%). mp 39–43 °C; $[\alpha]_D^{27}$ −132 (*c* 1.0, CH_2Cl_2); R_f 0.4 (petroleum ether/EtOAc, 4:1); IR $\tilde{\nu}_{max}$ 2928, 1750, 1263, 1101 cm^{-1}; ^{1}H NMR (500 MHz, $CDCl_3$) δ 5.20 (s, 1H), 4.83–4.79 (m, 1H), 4.08 (d, J = 7.8 Hz, 1H), 4.00 (ddd, J = 7.8, 4.8, 1.3 Hz, 1H), 3.50 (dddd, J = 15.2, 14.7, 7.8, 6.7 Hz, 1H), 2.42–2.29 (m, 2H); ^{13}C NMR (125 MHz, $CDCl_3$) δ 192.1, 124.2 (q, J = 279.1 Hz), 101.0 (br d, J = 1.5 Hz), 72.3, 67.7, 45.0 (q, J = 27.2 Hz), 31.4 (q, J = 2.4 Hz); ^{19}F NMR (471 MHz, $CDCl_3$) δ −72.3; HRMS (ESI-TOF) m/z: $[M-H]^-$ calcd for $C_7H_6F_3O_3^-$: 195.0269; found: 195.0274.

(3*R*,5*S*)-5-(Hydroxymethyl)-3-(trifluoromethyl)dihydrofuran-2(3*H*)-one (20). To a solution of **19** (320 mg, 1.6 mmol) in MeCN (7 mL) was added 30% H_2O_2 (0.83 mL, 8.2 mmol) and the mixture heated to 50 °C for 16 h. The reaction was quenched by the addition of Pt/C (5 mg) until the evolution of oxygen had ceased, and then 2 M HCl (2 mL) was added. The mixture was filtered through a pad of Celite, the filtrate concentrated under reduced pressure, and then the residue was purified by flash column chromatography (petroleum ether/EtOAc 2:1) to yield **20** as a colourless oil (154 mg, 51%). $[\alpha]_D^{28}$ +9 (*c* 0.5, CH_2Cl_2); R_f 0.3 (petroleum ether/EtOAc, 1:1); IR $\tilde{\nu}_{max}$ 3444, 2969, 1766, 1368, 1119 cm^{-1}; ^{1}H NMR (500 MHz, $CDCl_3$) δ 4.75–4.68 (m, 1H), 4.01 (dd, J = 12.4, 2.2 Hz, 1H), 3.69 (dd, J = 12.4, 2.6 Hz, 1H), 3.69–3.59 (m, 1H), 2.60–2.47 (m, 2H), 2.40 (br s, 1H); ^{13}C NMR (125 MHz, $CDCl_3$) δ 169.5,

124.0 (q, *J* = 279.0 Hz), 78.0, 64.0, 45.0 (q, *J* = 30.0 Hz), 25.0; 19F NMR (471 MHz, $CDCl_3$) δ −72.2 (d, *J* = 9.2 Hz). HRMS (ESI-TOF) m/z: $[M+H]^+$ calcd for $C_6H_8F_3O_3^+$: 185.0420; found: 185.0424. Spectral data was previously reported as a mixture of diastereomers [9].

(1*S*,5*R*)-3-(Trifluoromethyl)-6,8-dioxabicyclo[3.2.1]oct-2-en-4-one (21). To solution of enamine **15** (500 mg, 1.77 mmol) in dry MeCN (11 mL) was added $TMSCF_3$ (1.05 mL, 7.1 mmol) and diacetoxyiodobenzene (1.14 g, 3.6 mmol). The mixture was stirred for 5 min at rt and then 1,4-diazabicyclo[2.2.2]octane (397 mg, 3.6 mmol) and KF (411 mg, 7.1 mmol) were added, and the mixture was stirred for 90 min at rt. The mixture was quenched by the addition of 2 M HCl (25 mL) and the aqueous layer was extracted with EtOAc (4 × 20 mL). The combined organic layers were dried (Na_2SO_4), the volatiles removed under reduced pressure, and then the residue was purified by flash column chromatography (petroleum ether/EtOAc 6:1) to yield **21** as a colourless solid (145 mg, 42%). mp 41–42 °C; $[\alpha]_D^{28}$ −292 (*c* 1.0, CH_2Cl_2); R_f 0.3 (petroleum ether/EtOAc, 4:1); IR $\tilde{\nu}_{max}$ 2922, 1717, 1380, 1296 cm^{-1}; ^1H NMR (500 MHz, $CDCl_3$) δ 7.71–7.67 (m, 1H), 5.44 (s, 1H), 5.21–5.14 (m, 1H), 3.98 (dd, *J* = 7.4, 4.8 Hz, 1H), 3.87 (d, *J* = 7.4 Hz, 1H); ^{13}C NMR (125 MHz, $CDCl_3$) δ 182.9, 147.7 (q, *J* = 5.0 Hz), 128.3 (q, *J* = 31.7 Hz), 120.6 (q, *J* = 273.8 Hz), 100.9 (br d, *J* = 0.9 Hz) , 71.3, 66.3; ^{19}F NMR (471 MHz, $CDCl_3$) δ −69.5; HRMS (ESI-TOF) m/z: $[M-H]^-$ calcd for $C_7H_4F_3O_3^-$: 193.0113 ; found: 193.0109.

(4*R*)-4-(Hydroxymethyl)-1-(trifluoromethyl)-3,6-dioxabicyclo[3.1.0]hexan-2-one (22). To a solution of **21** (85 mg, 0.437 mmol) in MeCN (2 mL) was added 30% H_2O_2 (0.225 mL, 2.18 mmol) and the mixture heated to 50 °C for 16 h. The reaction mixture was quenched by the addition of Pt/C (5 mg), and once the evolution of oxygen had ceased, 2 M HCl (0.5 mL) was added. The mixture filtered through a pad of Celite, the filtrate was concentrated under reduced pressure, and the residue was purified by flash column chromatography (petroleum ether/EtOAc, 2:1) to yield **22** as a colourless solid (58 mg, 67%, dr 2:1). mp 55–57 °C; IR $\tilde{\nu}_{max}$ 3325, 2983, 2883, 1456, 1382 cm^{-1}; Major diastereomer: ^1H NMR (500 MHz, $CDCl_3$) δ 4.67 (dd, *J* = 3.0, 2.1 Hz, 1H), 4.45 (app. s, 1H), 4.07 (dd, *J* = 12.6, 3.0 Hz, 1H), 3.93 (dd, *J* = 12.6, 2.1 Hz, 1H), 2.15 (br s, 1H); ^{13}C NMR (125 MHz, $CDCl_3$) δ 164.7, 119.8 (q, *J* = 274.9 Hz), 78.1, 60.9, 60.3, 55.7 (q, *J* = 42.6 Hz); ^{19}F NMR (471 MHz, $CDCl_3$) δ −74.6; Minor diastereomer (partial): ^1H NMR (500 MHz, $CDCl_3$) δ 4.56 (br dd, *J* = 9.7, 7.0 Hz, 1H), 4.25–4.17 (m, 2H), 4.05 (br s, 1H); ^{13}C NMR (125 MHz, $CDCl_3$) δ 160.2, 65.7, 63.3, 54.5 (q, *J* = 38.0 Hz); ^{19}F NMR (471 MHz, $CDCl_3$) δ −74.6; HRMS (ESI-TOF) m/z: $[M+H]^+$ calcd for $C_6H_6F_3O_4^+$: 199.0213; found: 199.0227.

References

1. Sarotti, A. M.; Spanevello, R. A.; Suárez, A. G. *Tetrahedron Lett*. **2011**, *52* (32), 4145-4148.
2. Podversnik, H.; Curtis, I.; Pieterse, E.; Jevric, M.; Sumby, C. J.; Greatrex, B. W. *Tetrahedron Lett.* **2023**, *129*, 154755.
3. Kawai, T.; Isobe, M.; Peters, S. C. *Aust. J. Chem.* **1995**, *48* (1), 115-131.
4. Matoušek, V.; Pietrasiak, E.; Schwenk, R.; Togni, A. *J. Org. Chem.* **2013**, *78* (13), 6763-6768.
5. Nishikawa, T.; Asai, M.; Ohyabu, N.; Yamamoto, N.; Fukuda, Y.; Isobe, M. *Tetrahedron* **2001**, *57* (18), 3875-3883.
6. Goto, K.; Ideo, H.; Tsuchida, A.; Hirose, Y.; Maruyama, I.; Noma, S.; Shirai, T.; Amano, J.; Mizuno, M.; Matsuda, A. *Bioorg. Med. Chem.* **2018**, *26* (13), 3763-3772.
7. Flores, R.; Rustullet, A.; Alibés, R.; Álvarez-Larena, A.; de March, P.; Figueredo, M.; Font, J. *J. Org. Chem.* **2011**, *76* (13), 5369-5383.
8. Ellwood, A. R.; Mortimer, A. J. P.; Goodman, J. M.; Porter, M. J. *Org. Biomol. Chem.* **2013**, *11* (43), 7530-7539.
9. Zhang, X.; Qing, F.-L.; Yu, Y. *J. Org. Chem.* **2000**, 65, 7075-7082.

^{1}H (500 MHz, $CDCl_3$), ^{13}C (125 MHz, $CDCl_3$) and ^{19}F NMR (471 MHz, $CDCl_3$) spectra for all compounds

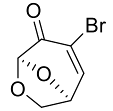

7b

^{1}H NMR spectrum

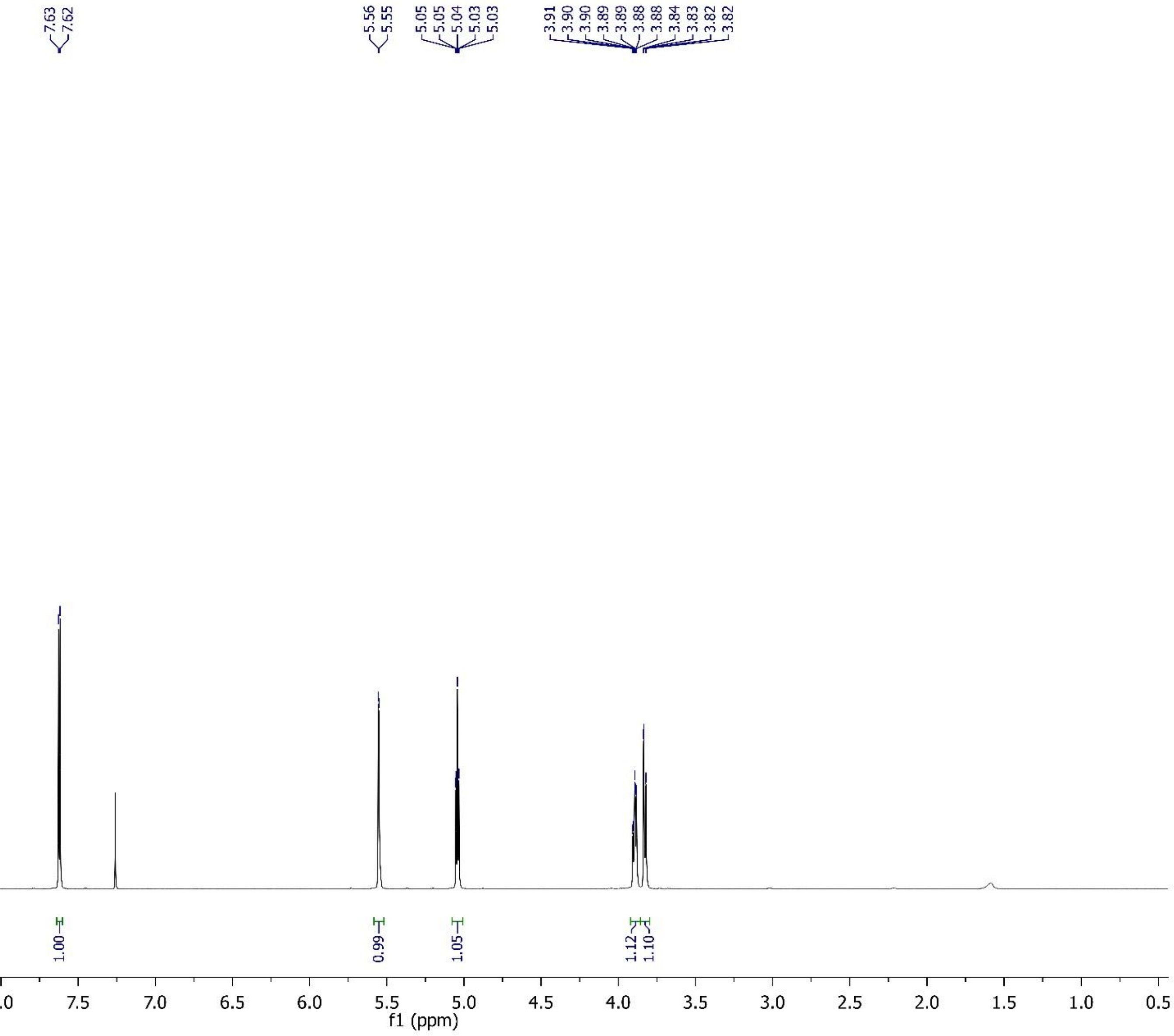

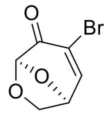

7b

^{13}C NMR spectrum

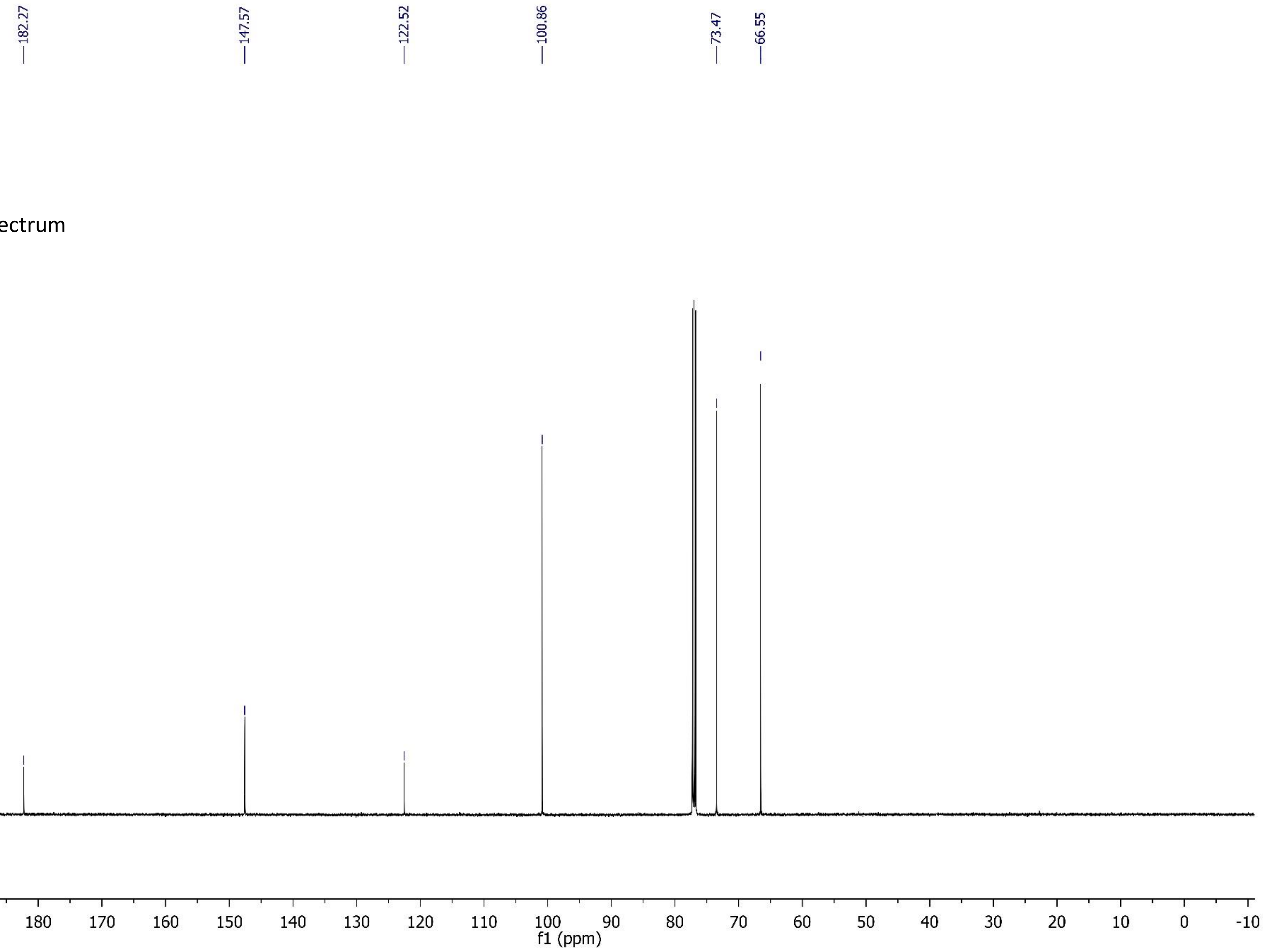

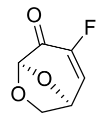

7c

^{1}H NMR spectrum

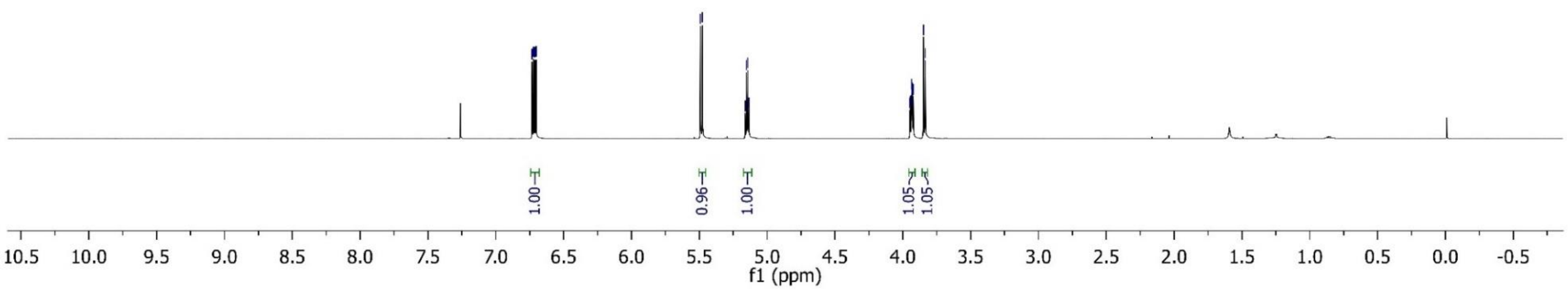

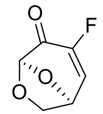

7c

^{13}C NMR spectrum

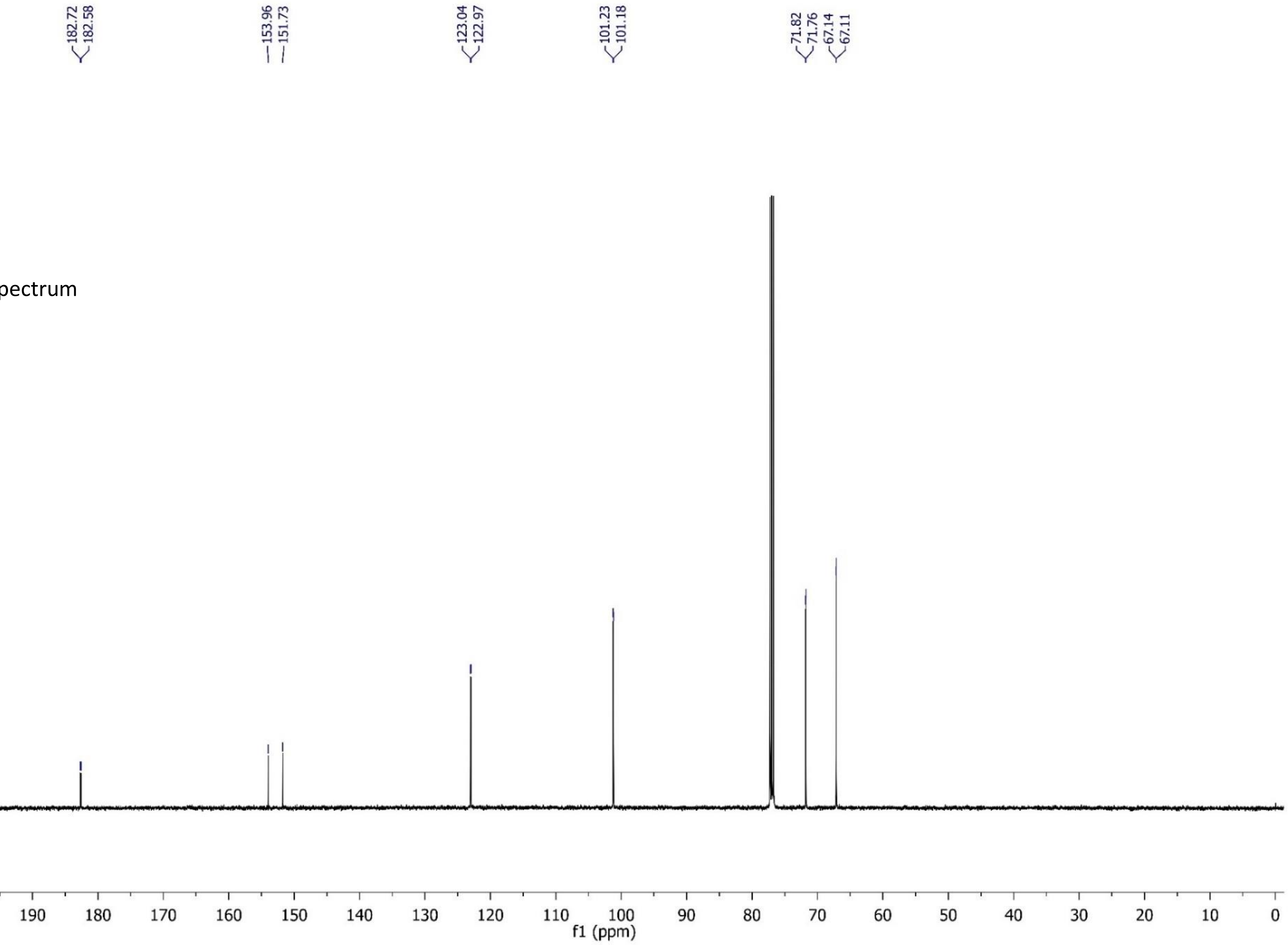

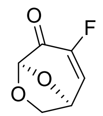

7c

^{19}F NMR spectrum

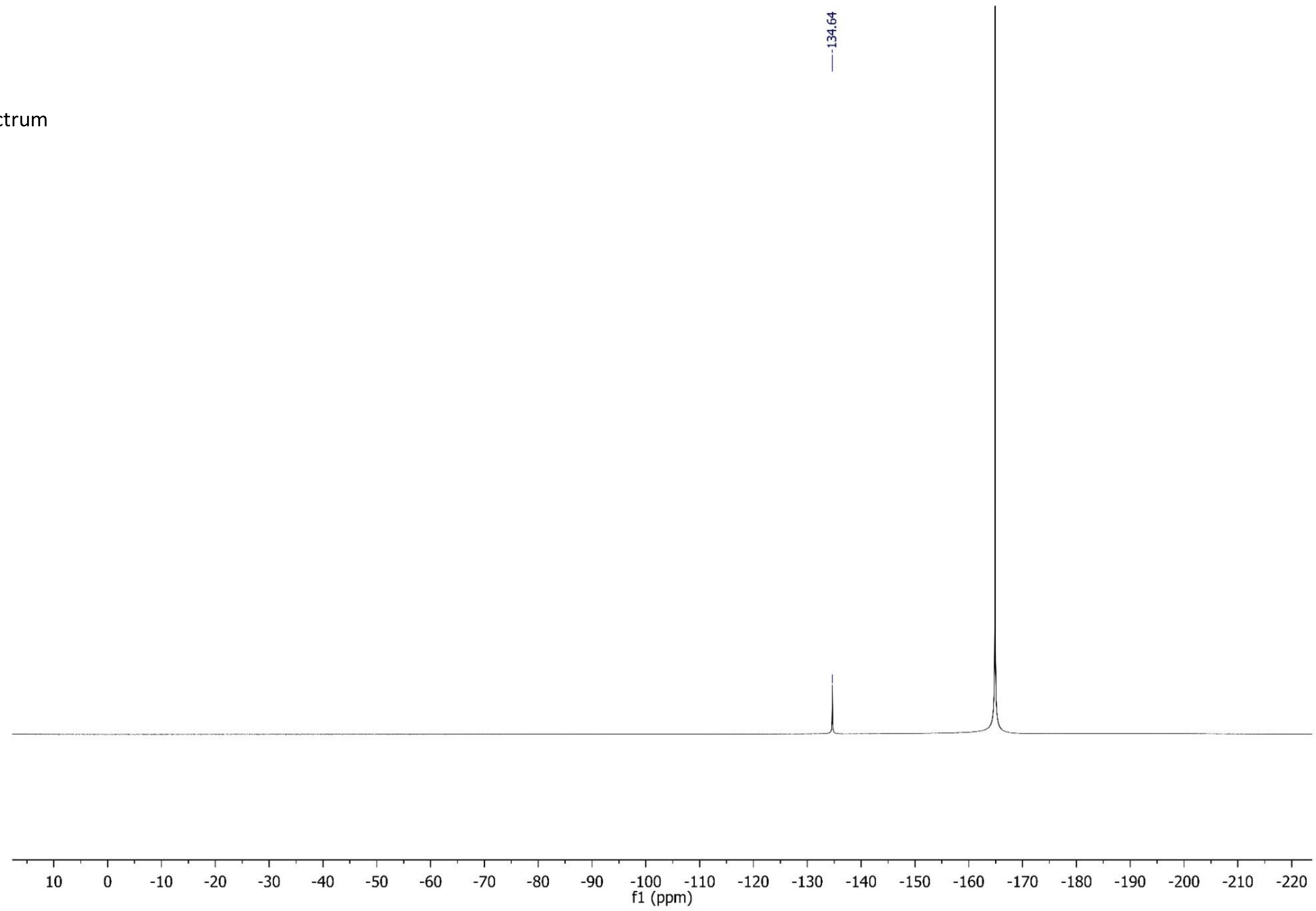

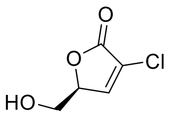

8a

^{1}H NMR spectrum

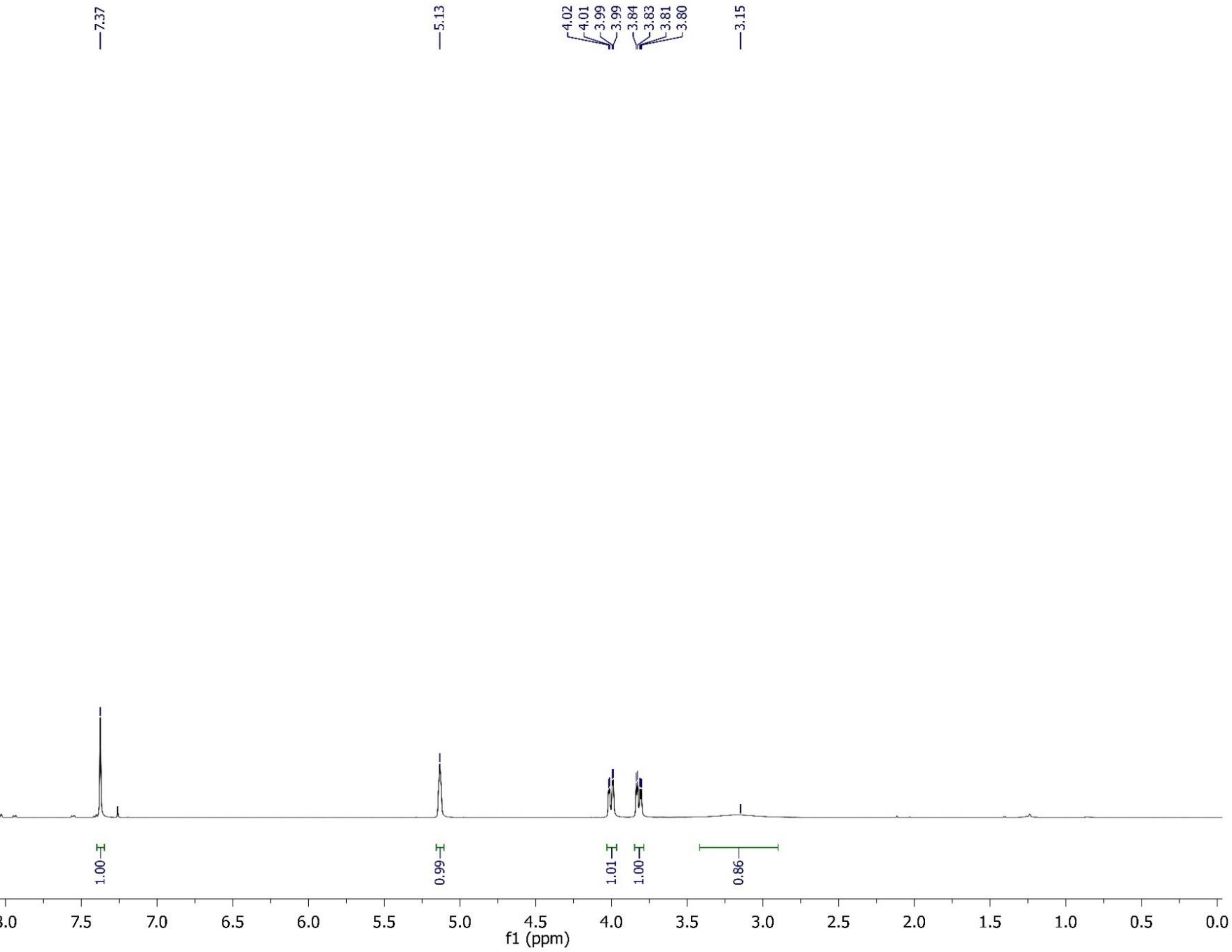

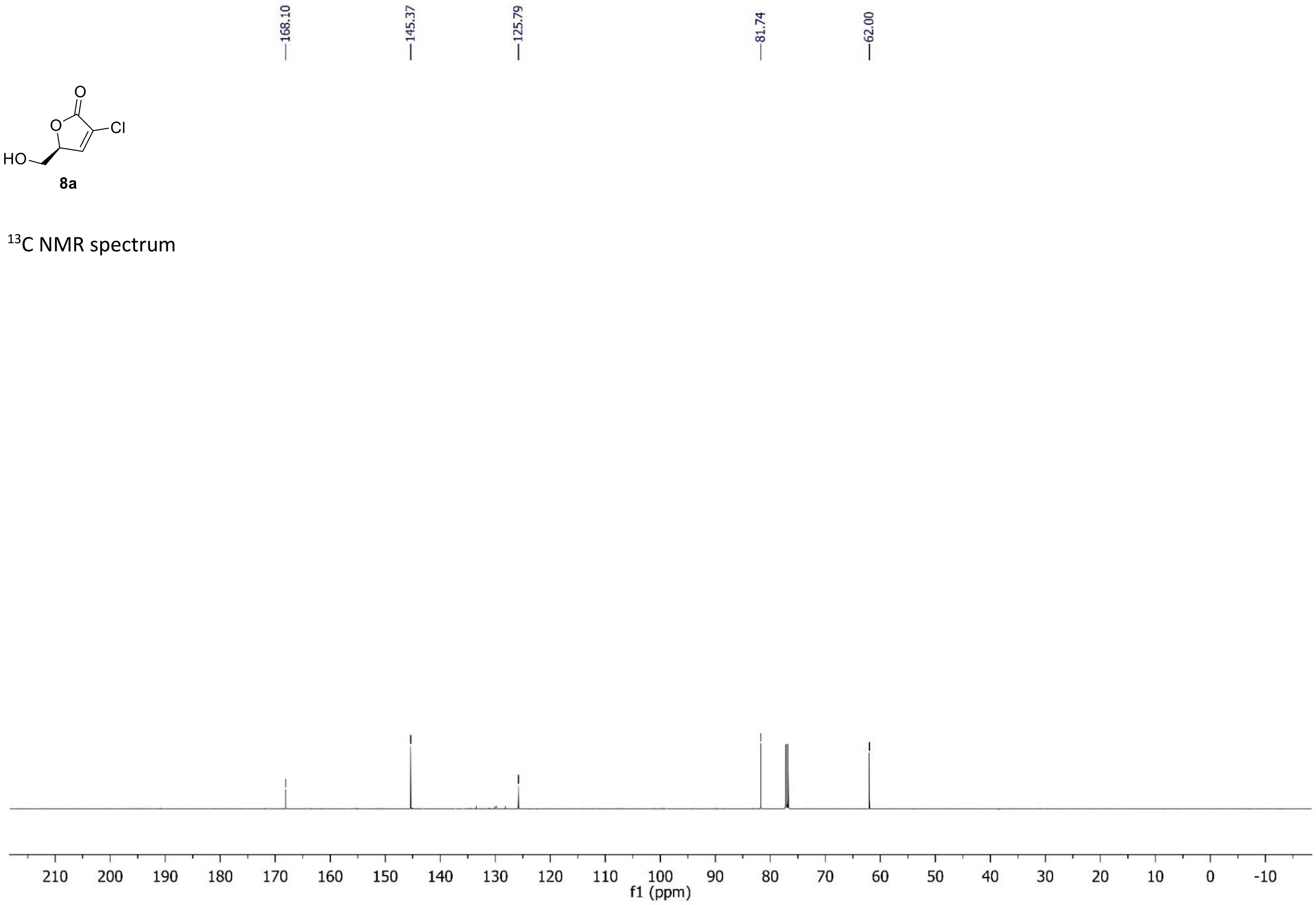

8a

^{13}C NMR spectrum

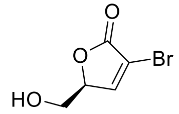

8b

^{1}H NMR spectrum

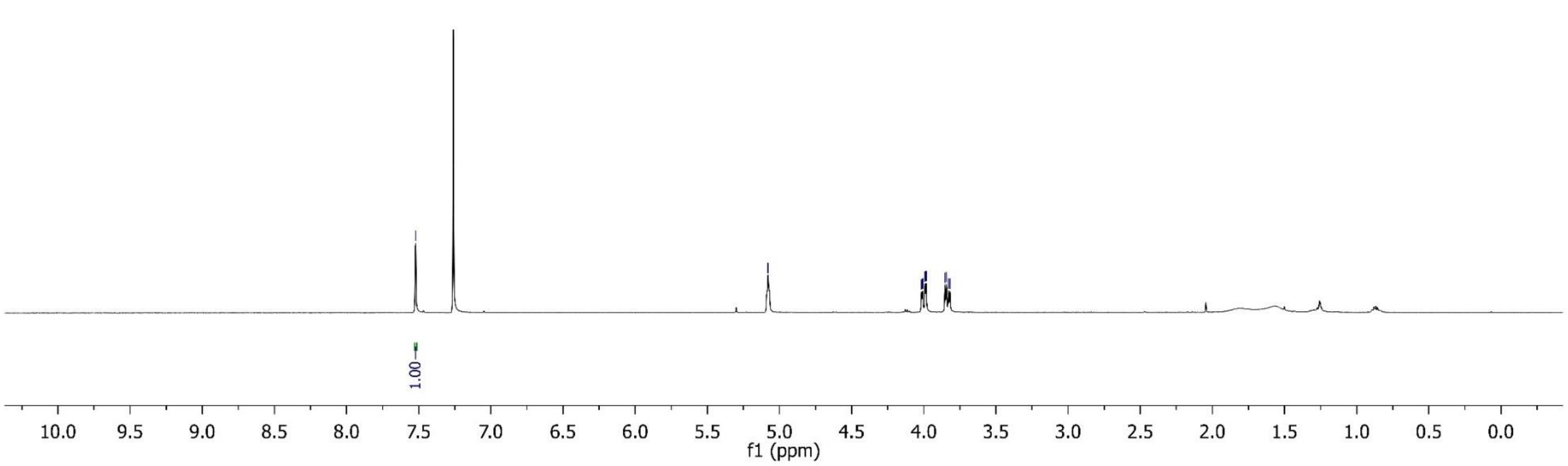

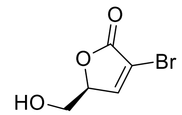

8b

^{13}C NMR spectrum

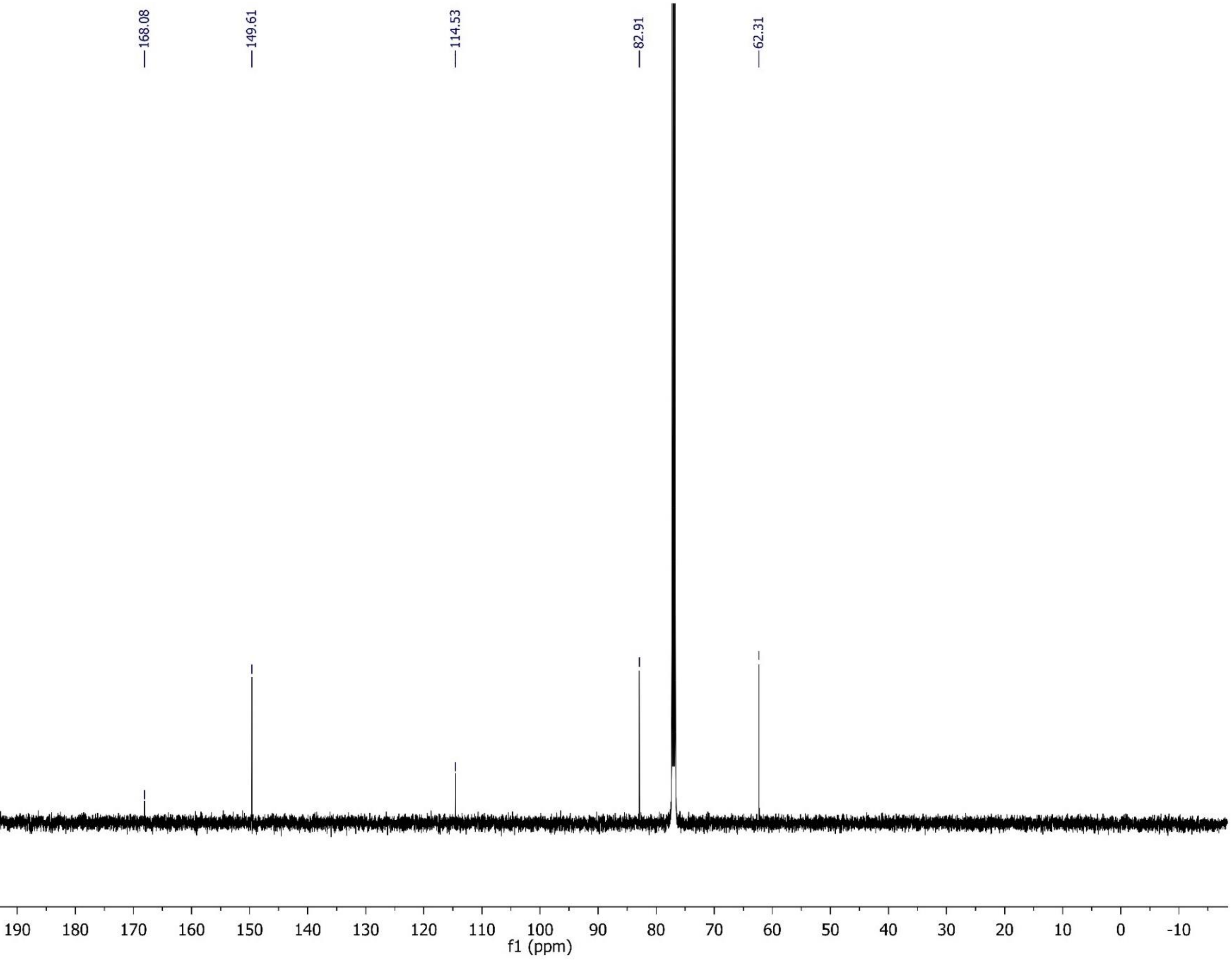

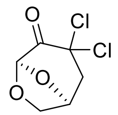

10a

^{1}H NMR spectrum

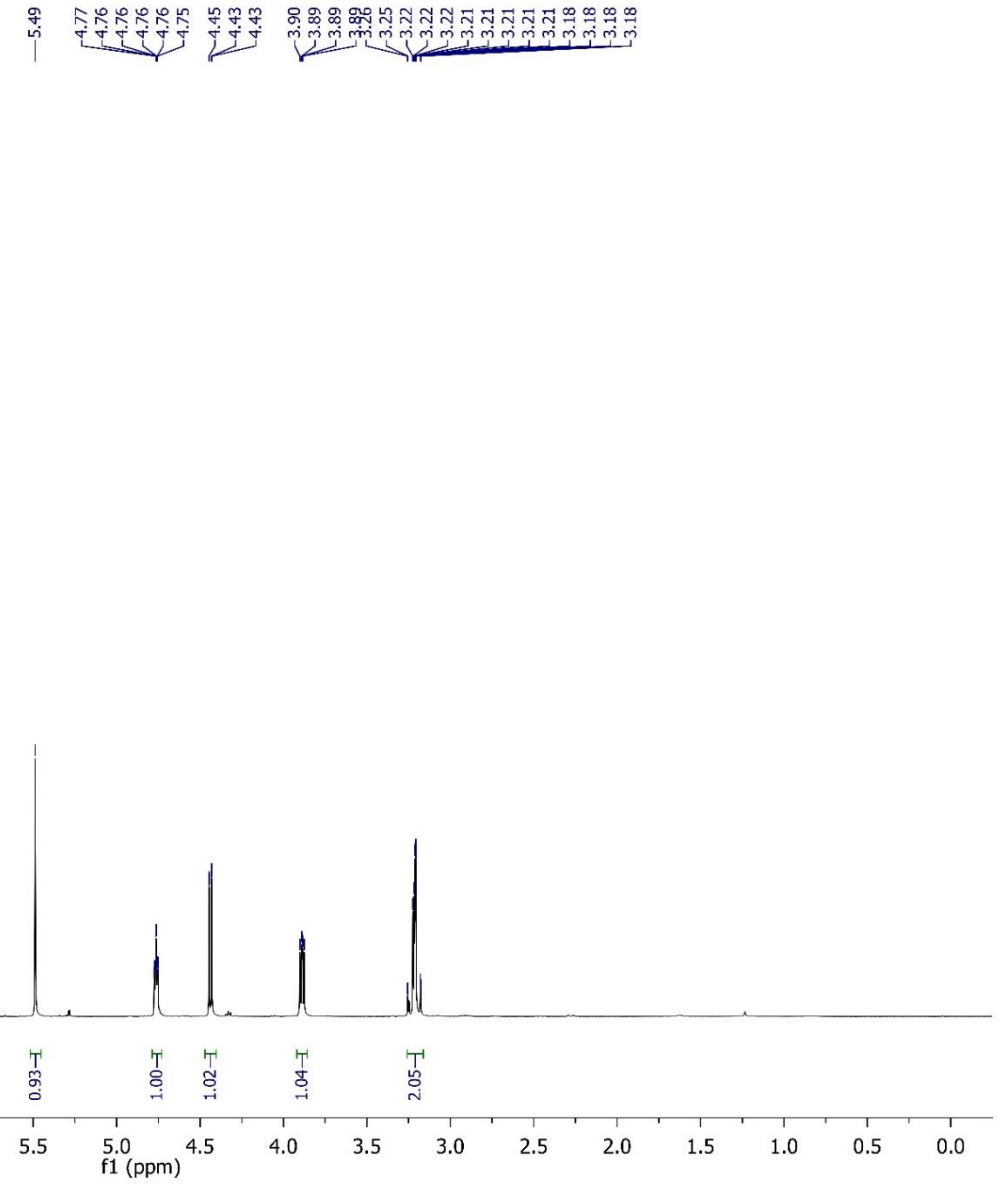

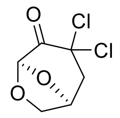

10a

^{13}C NMR spectrum

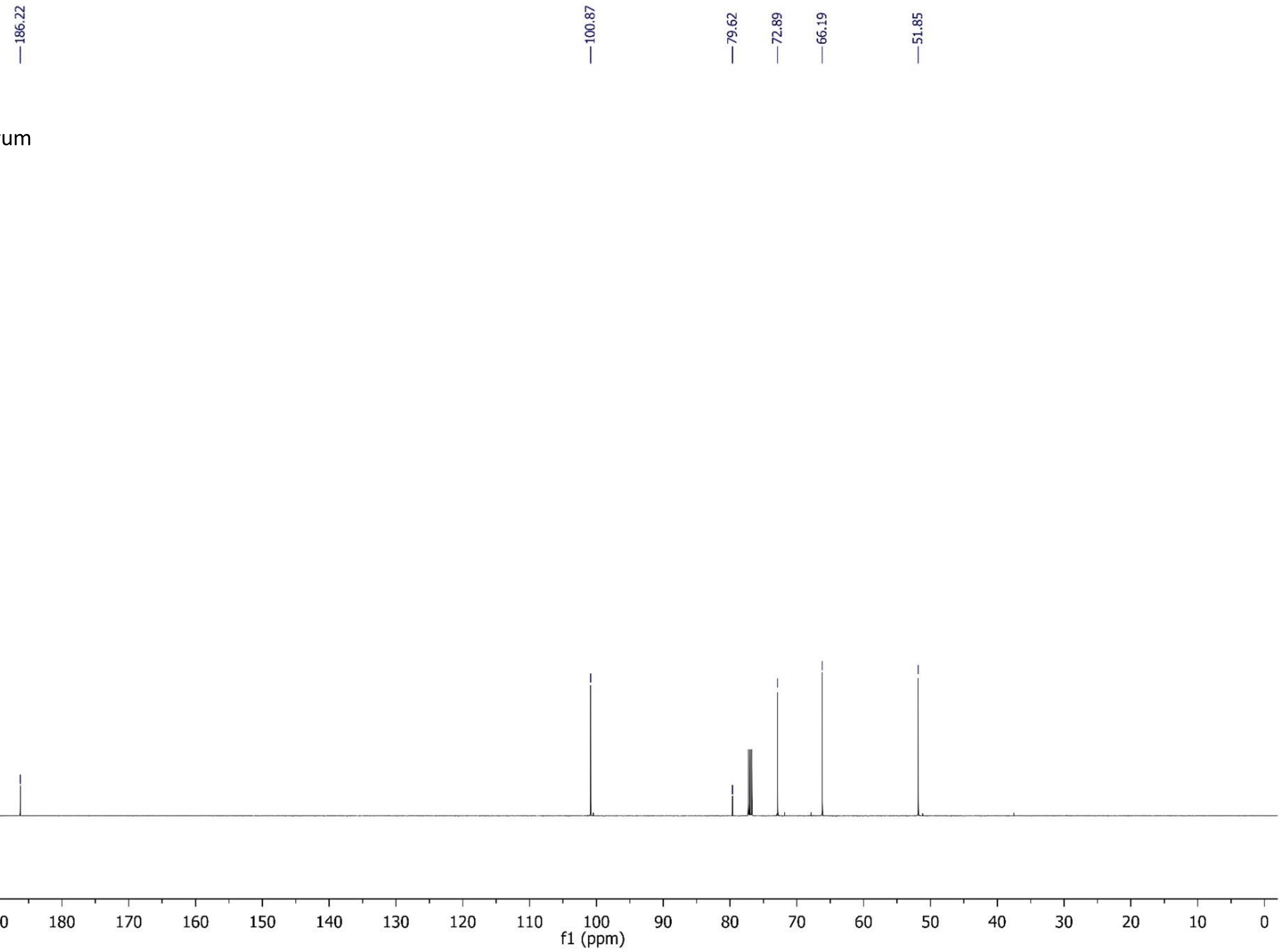

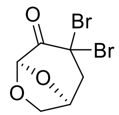

10b

^{1}H NMR spectrum

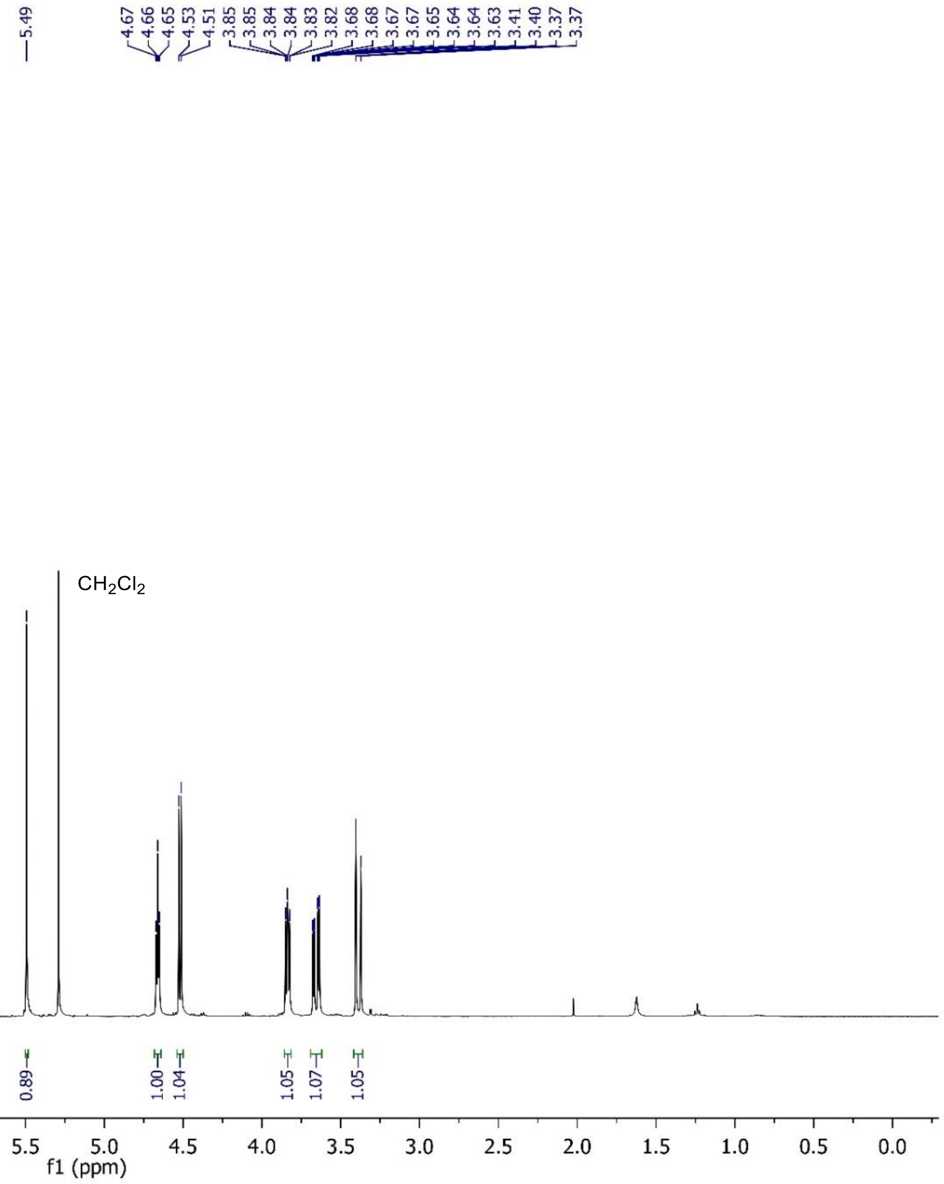

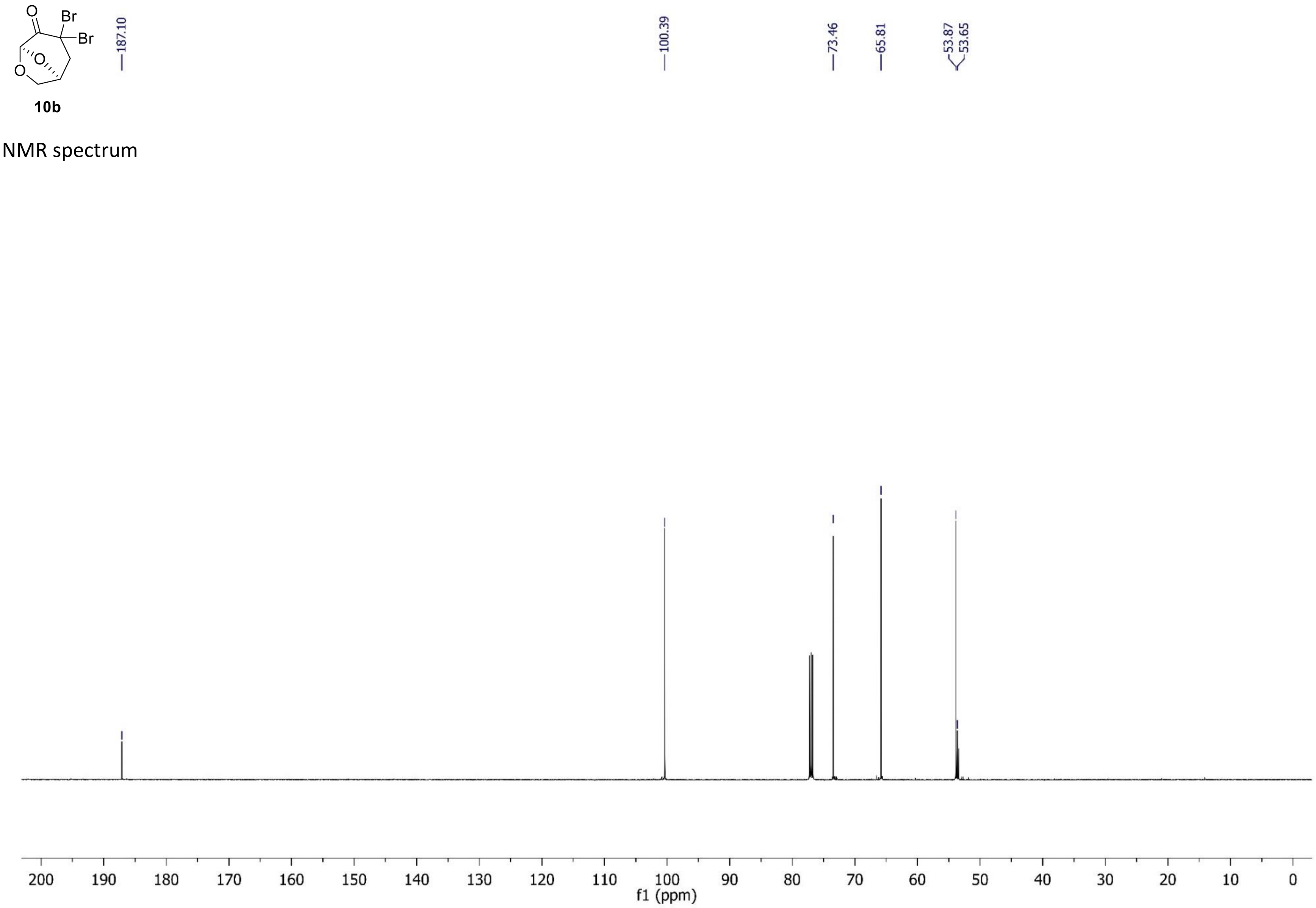

10b

^{13}C NMR spectrum

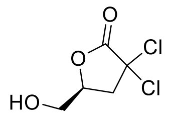

11a

^{1}H NMR spectrum

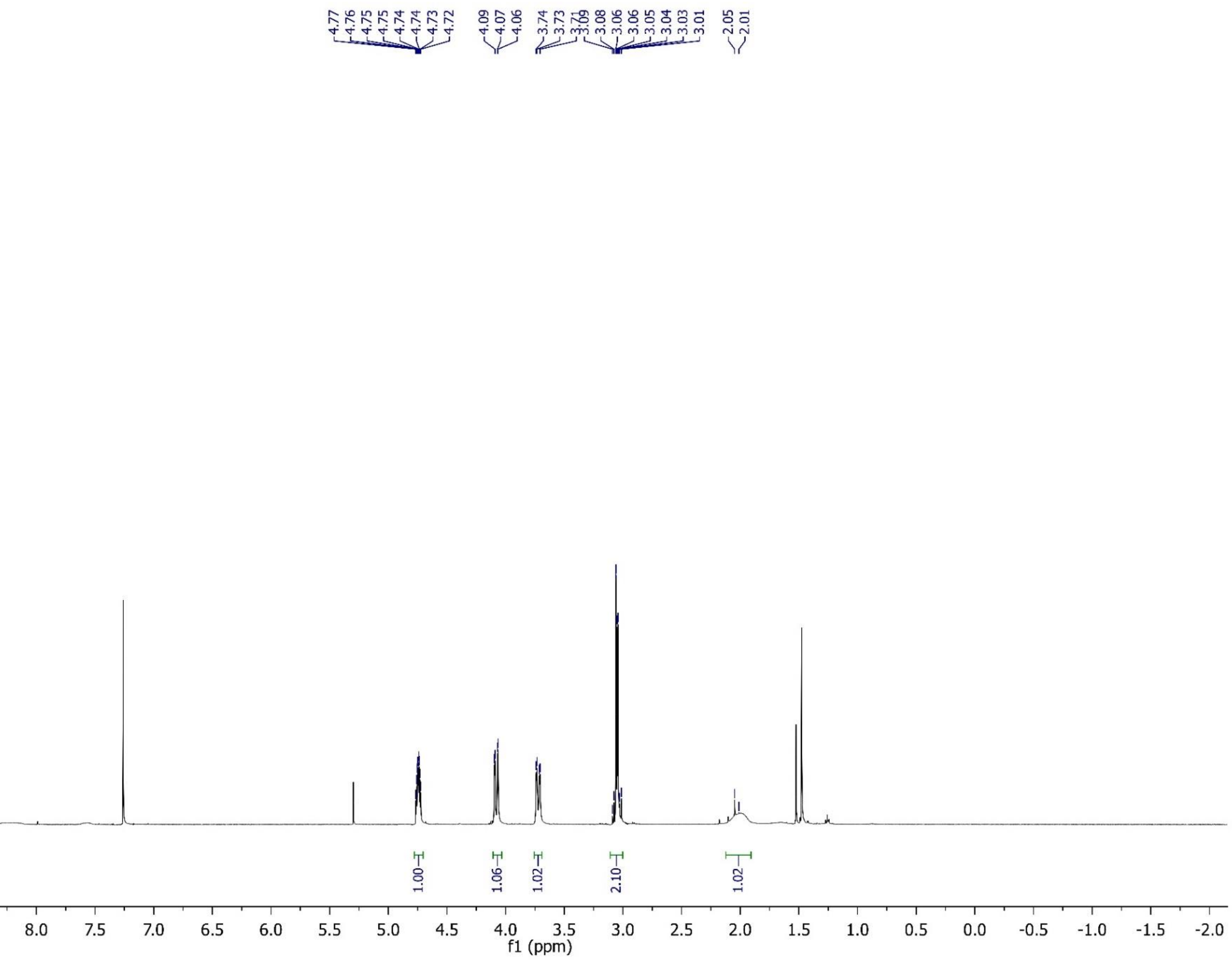

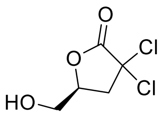

11a

^{13}C NMR spectrum

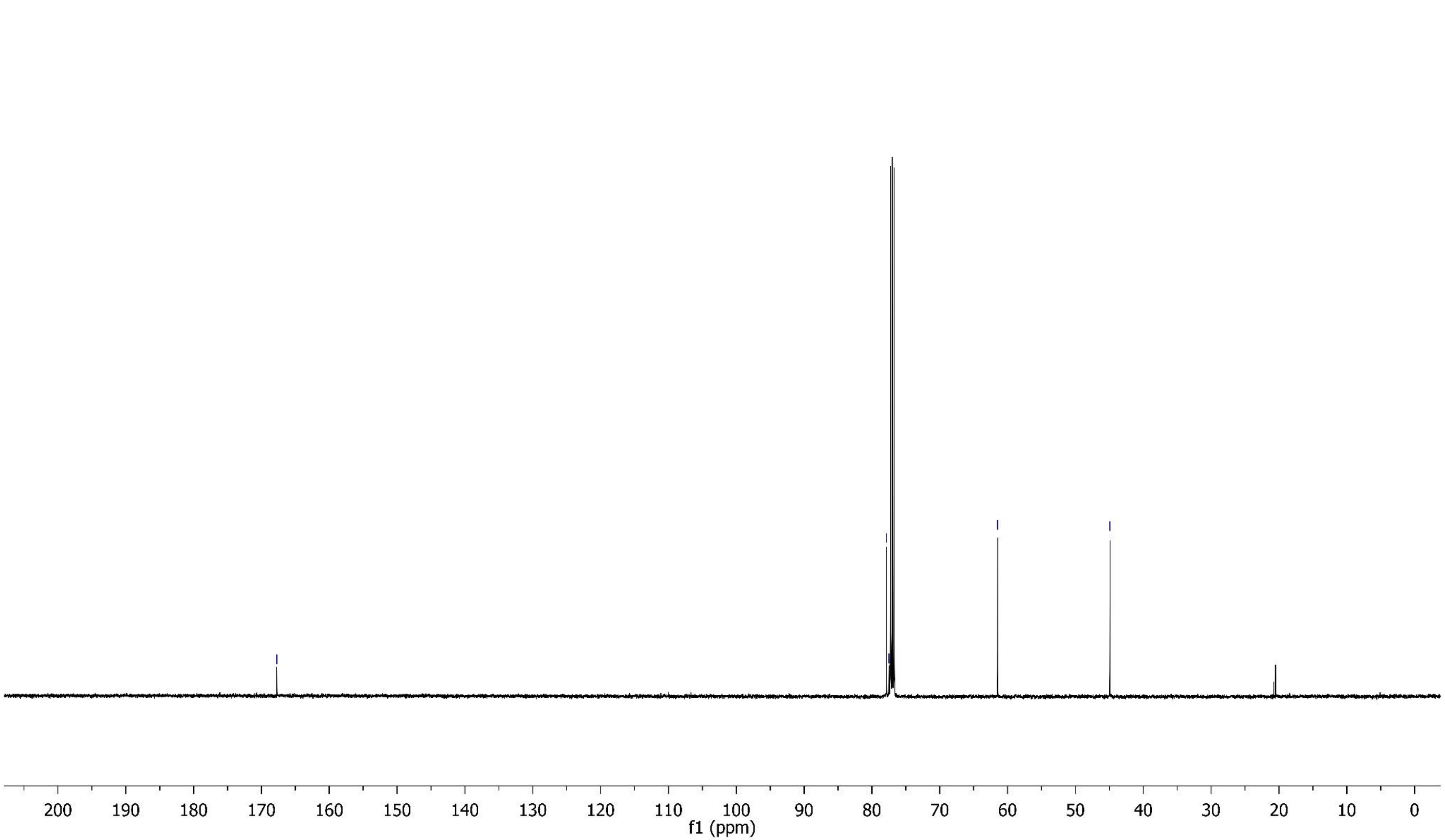

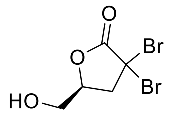

11b

^{1}H NMR spectrum

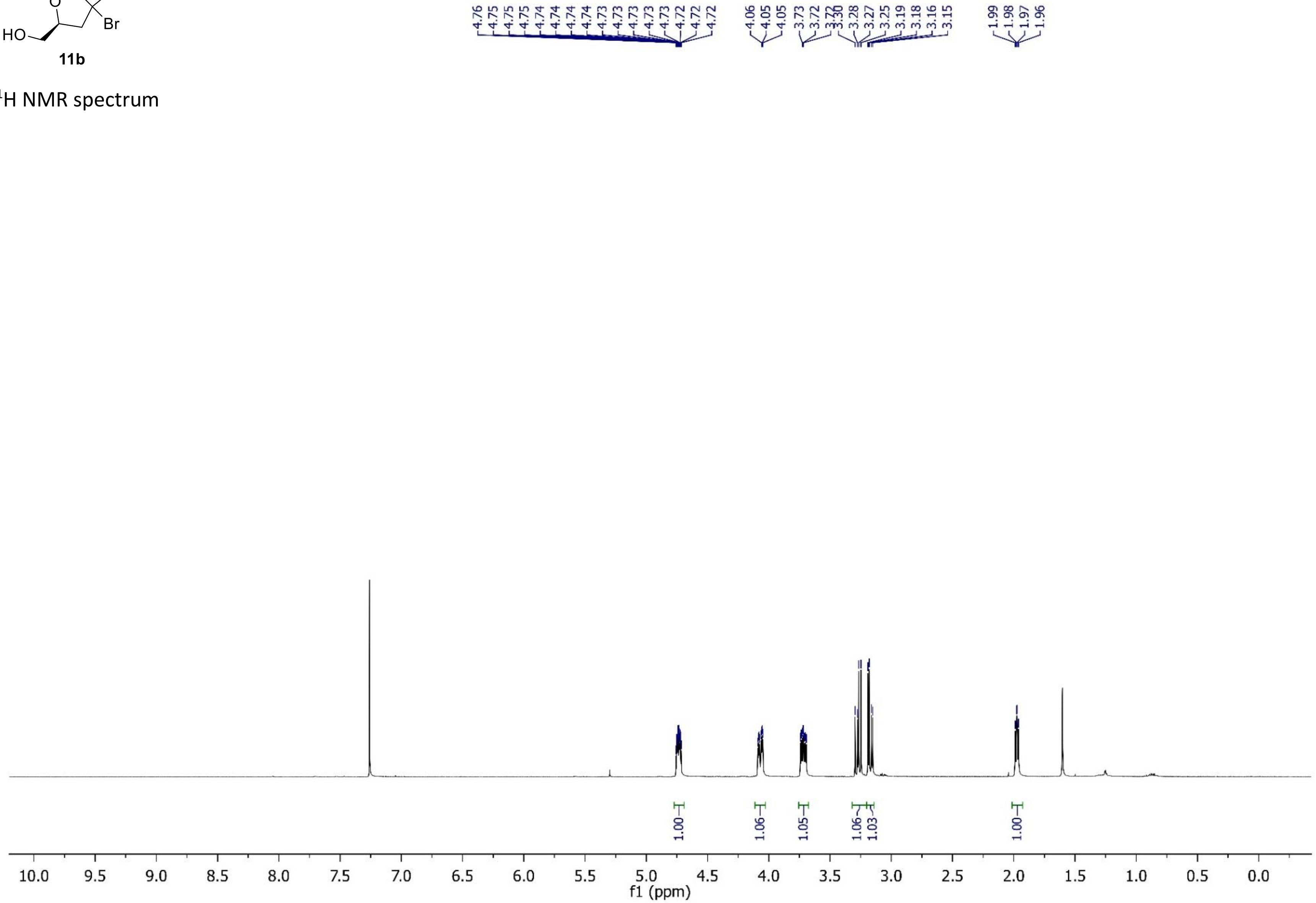

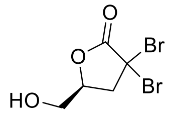

11b

^{13}C NMR spectrum

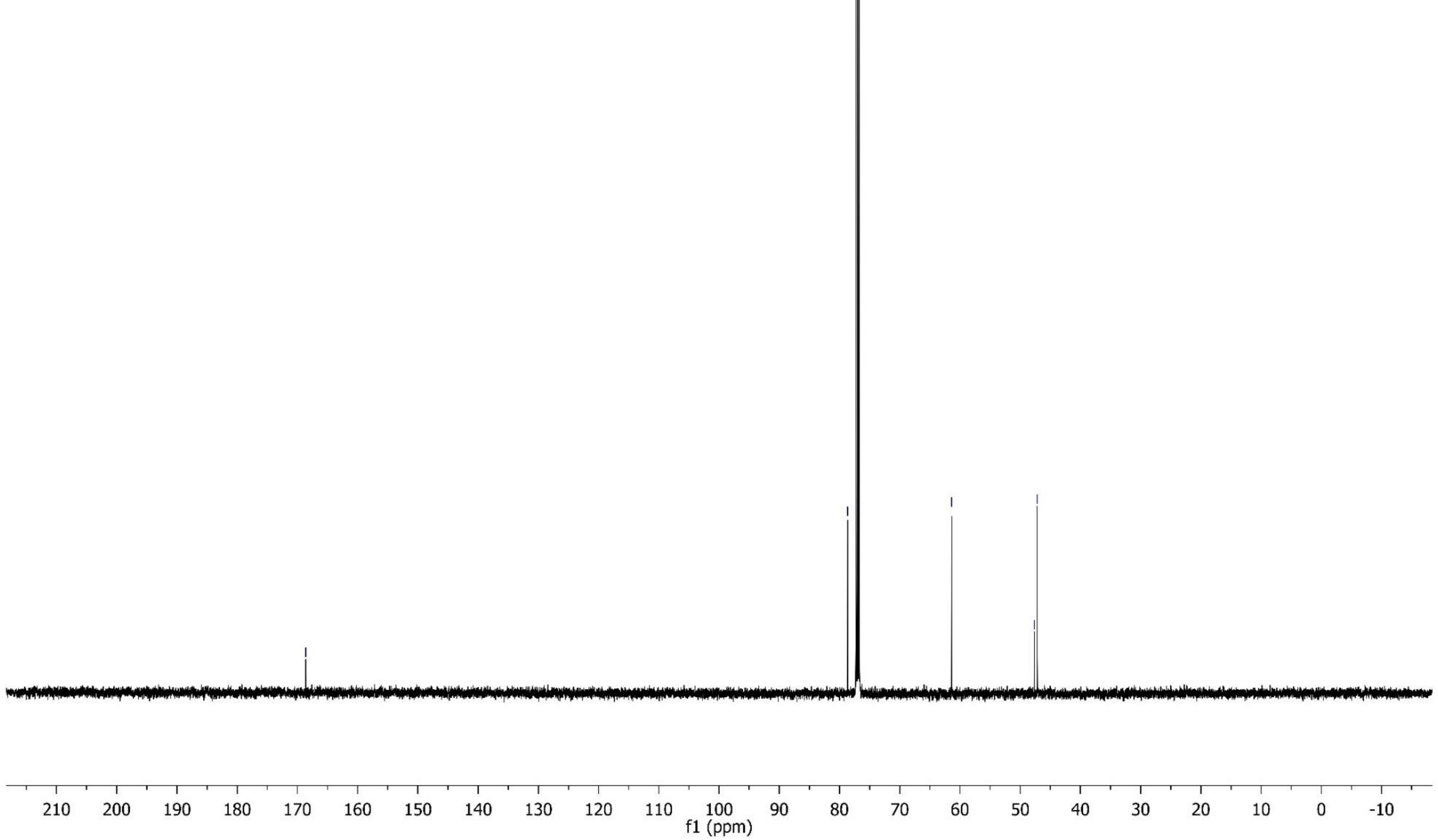

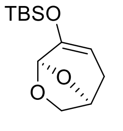

12

^{1}H NMR spectrum

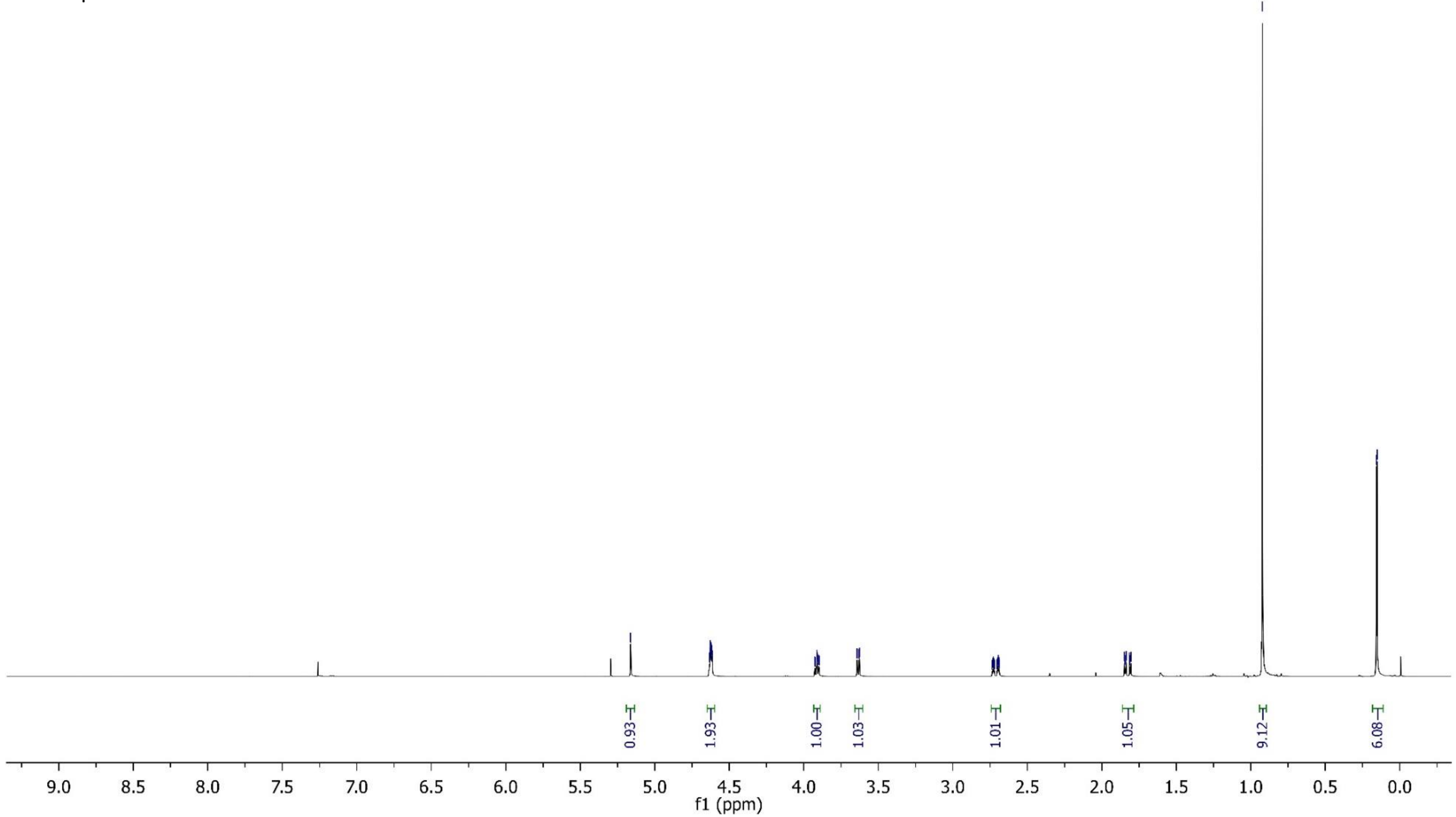

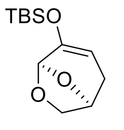

12

^{13}C NMR spectrum

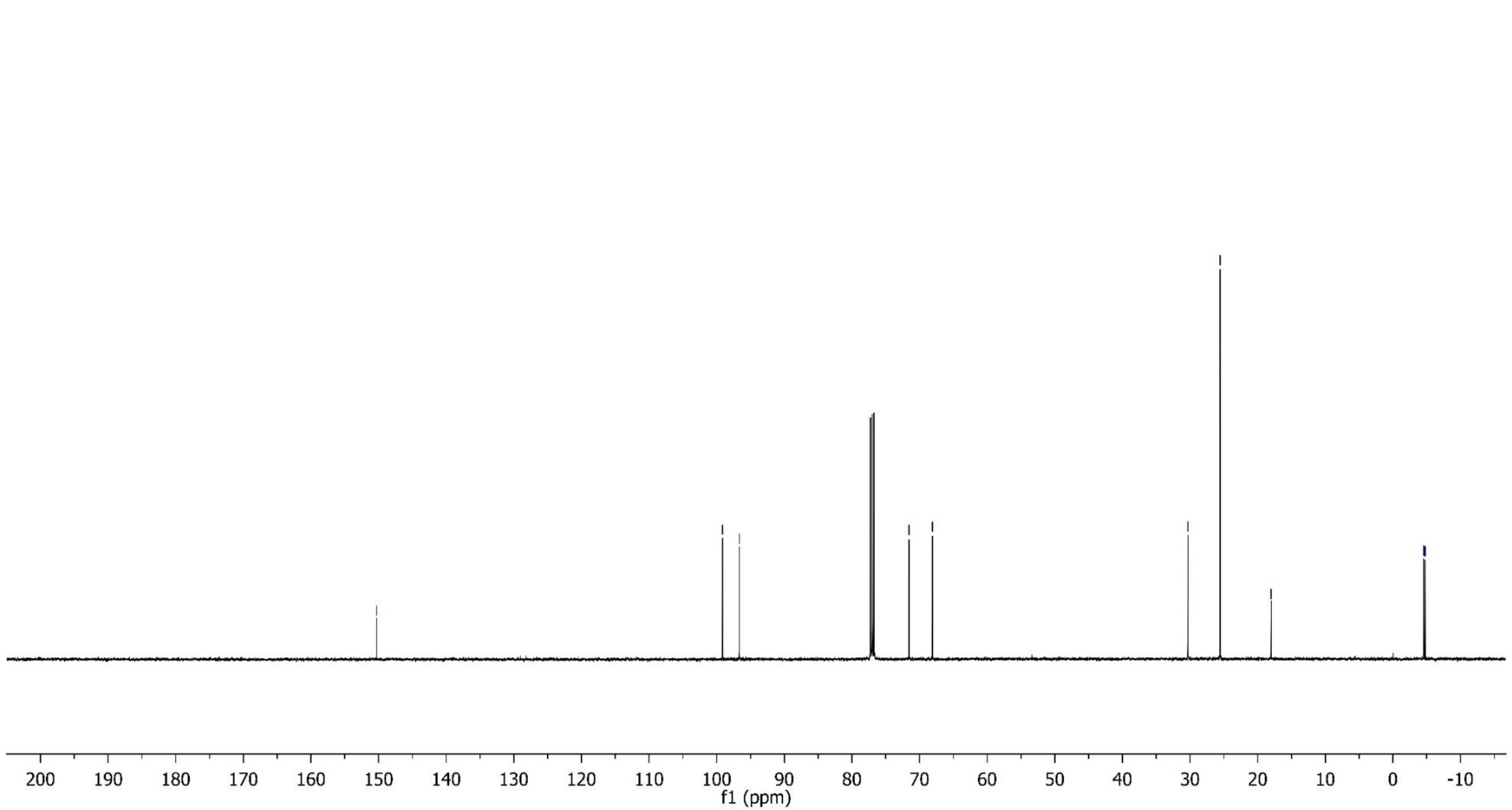

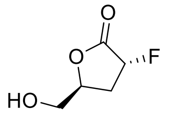

14

^{1}H NMR spectrum

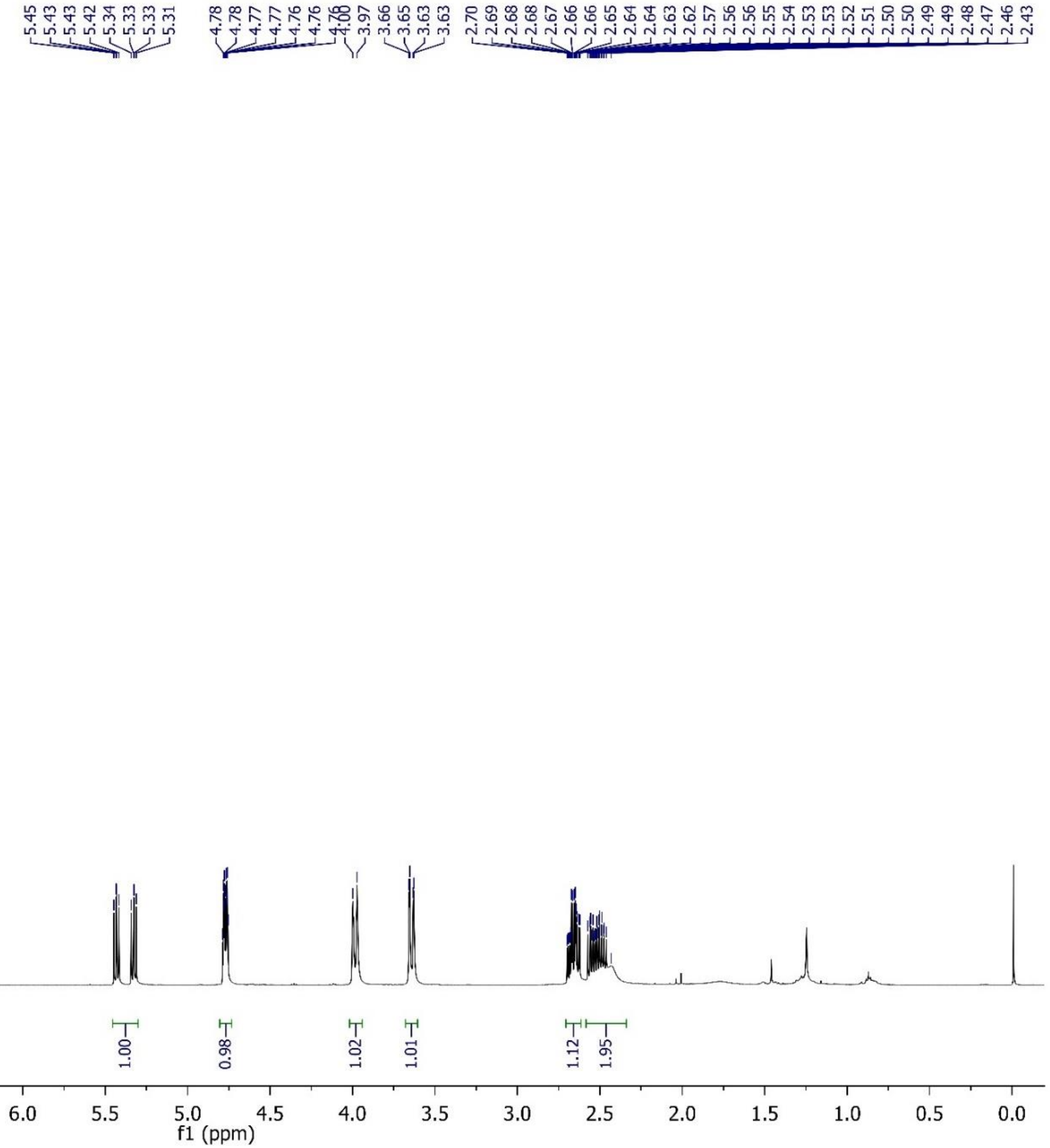

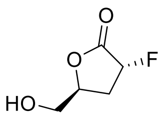

14

^{13}C NMR spectrum

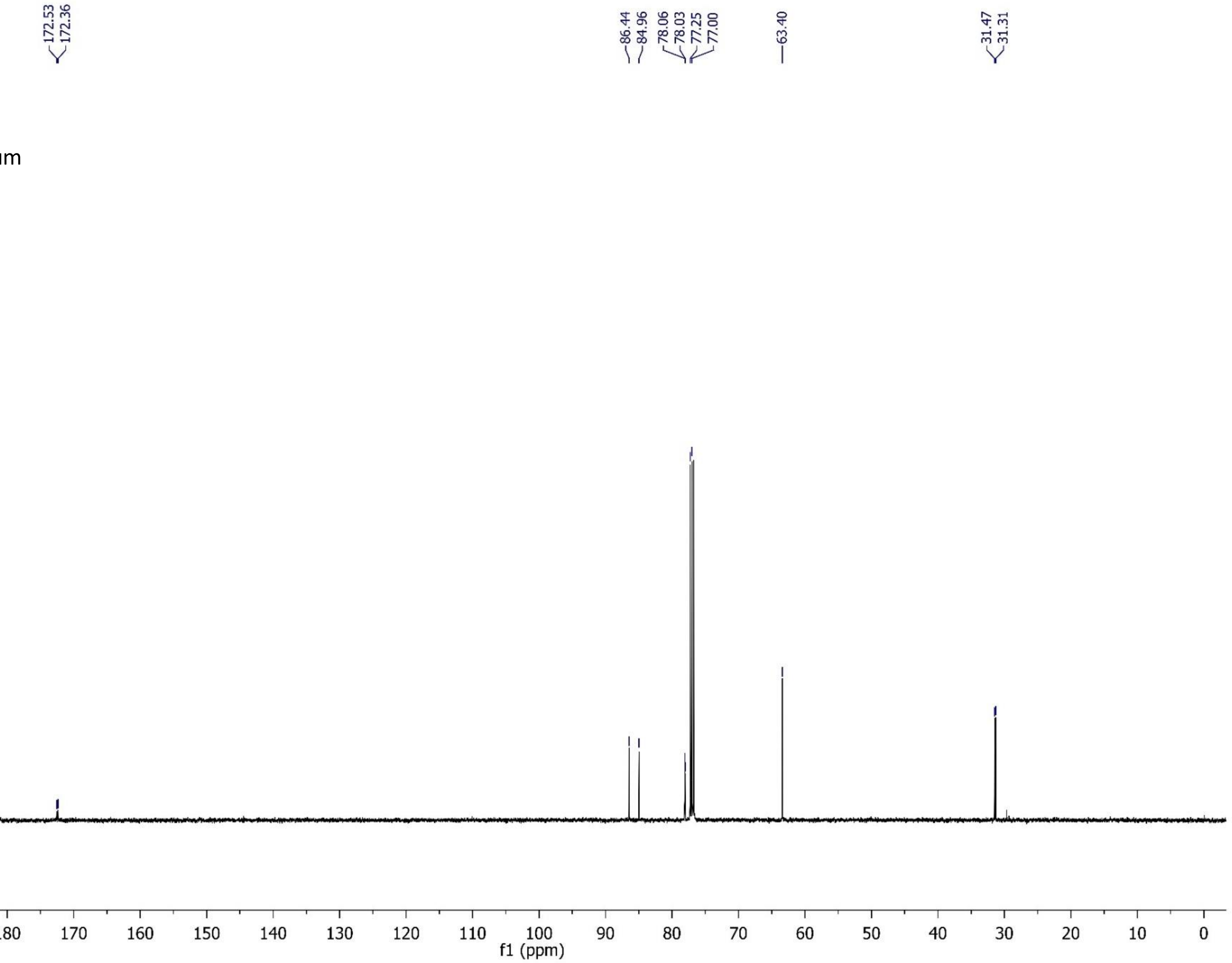

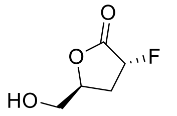

14

^{19}F NMR spectrum

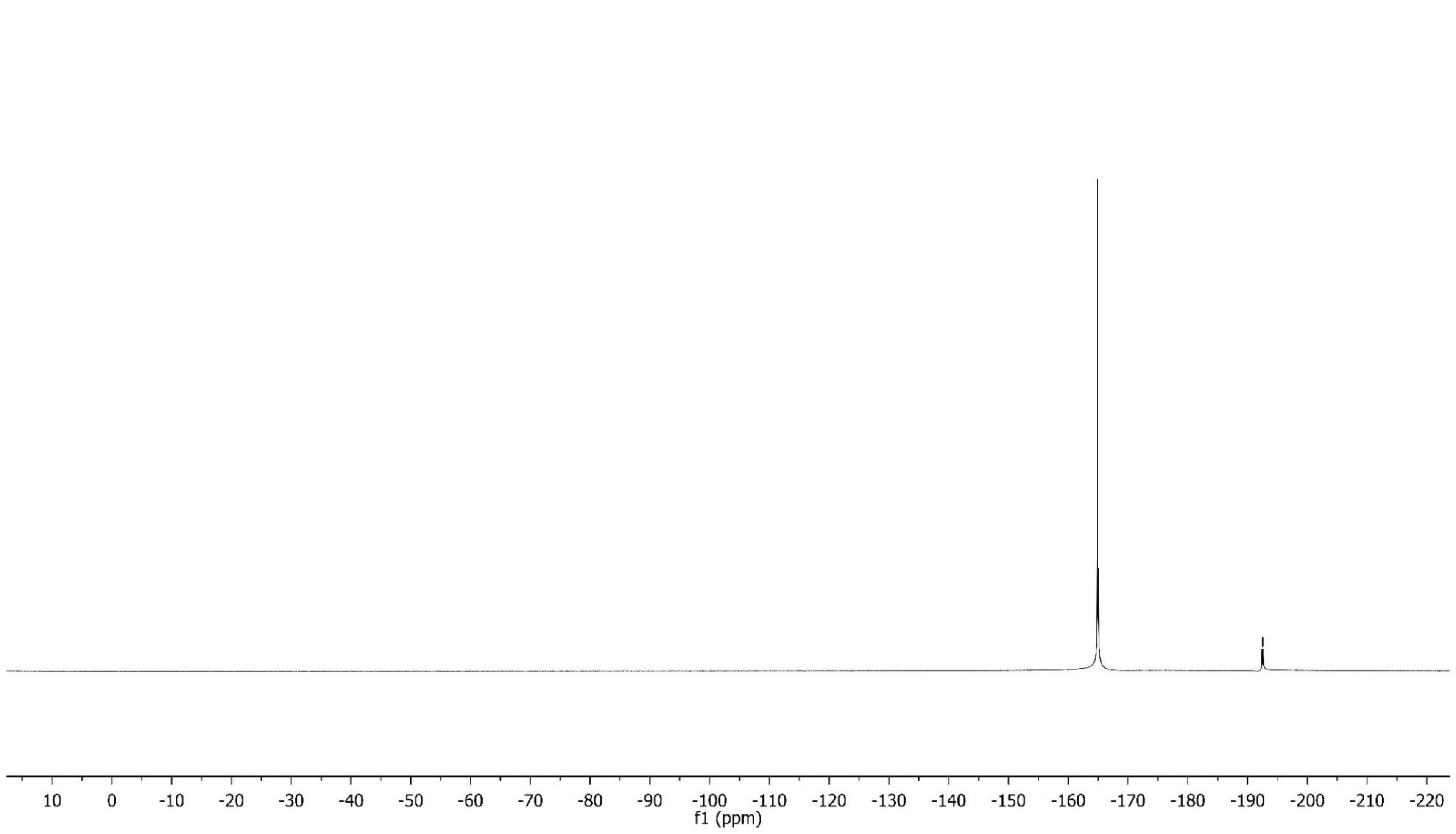

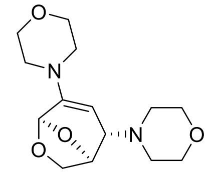

15

^{1}H NMR spectrum

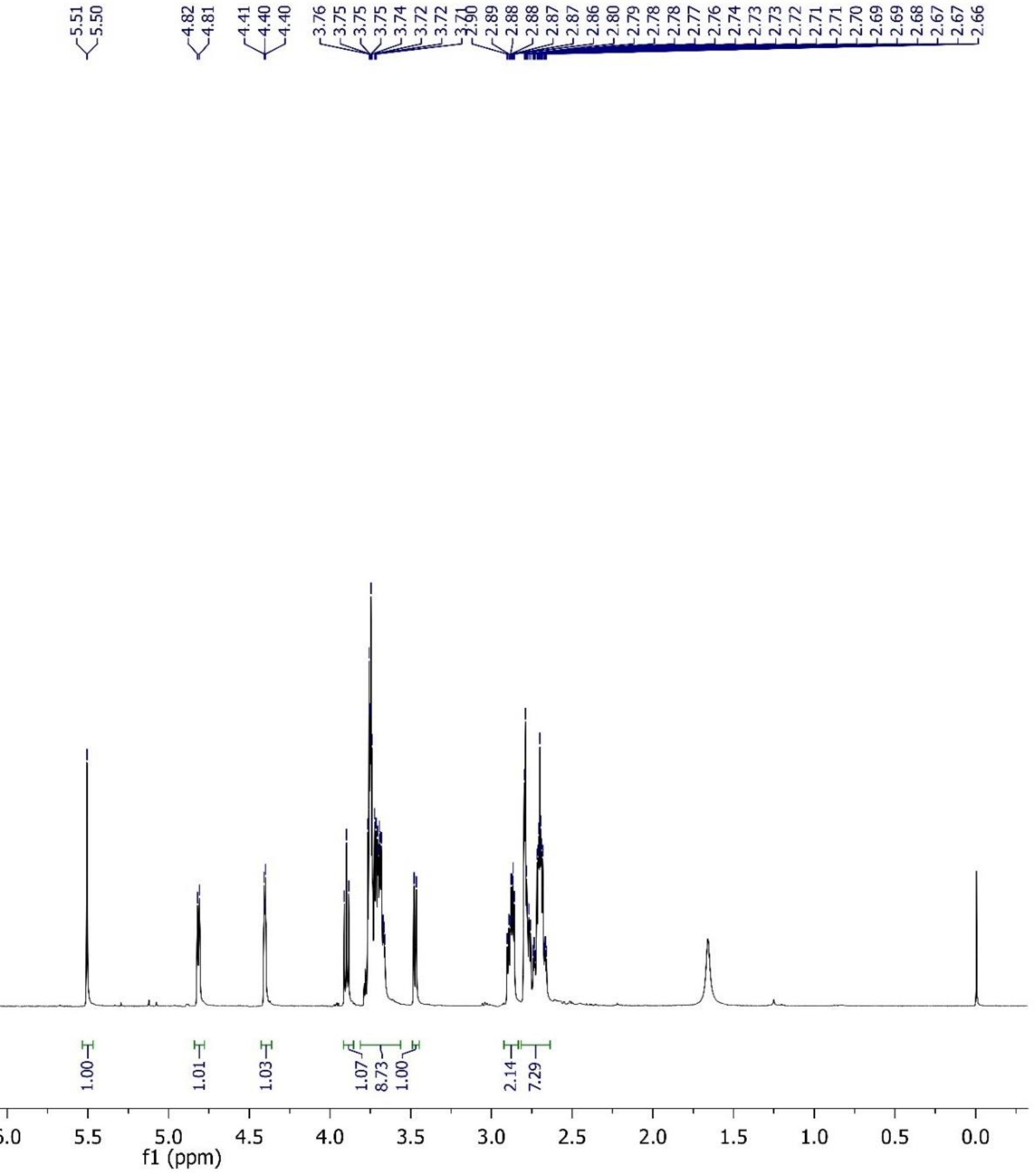

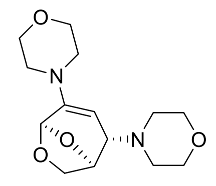

15

^{13}C NMR spectrum

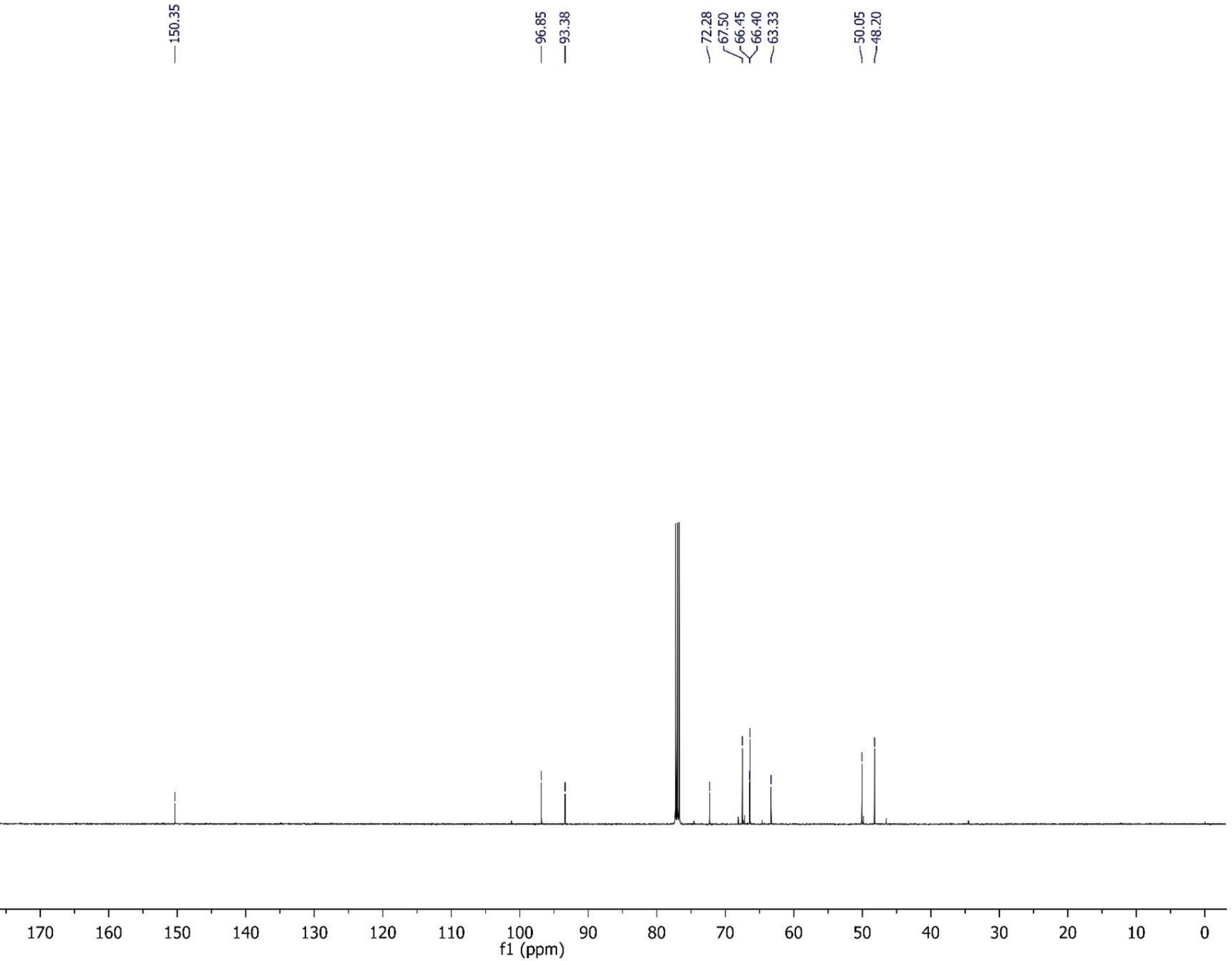

s

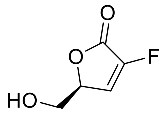

17

^{1}H NMR spectrum

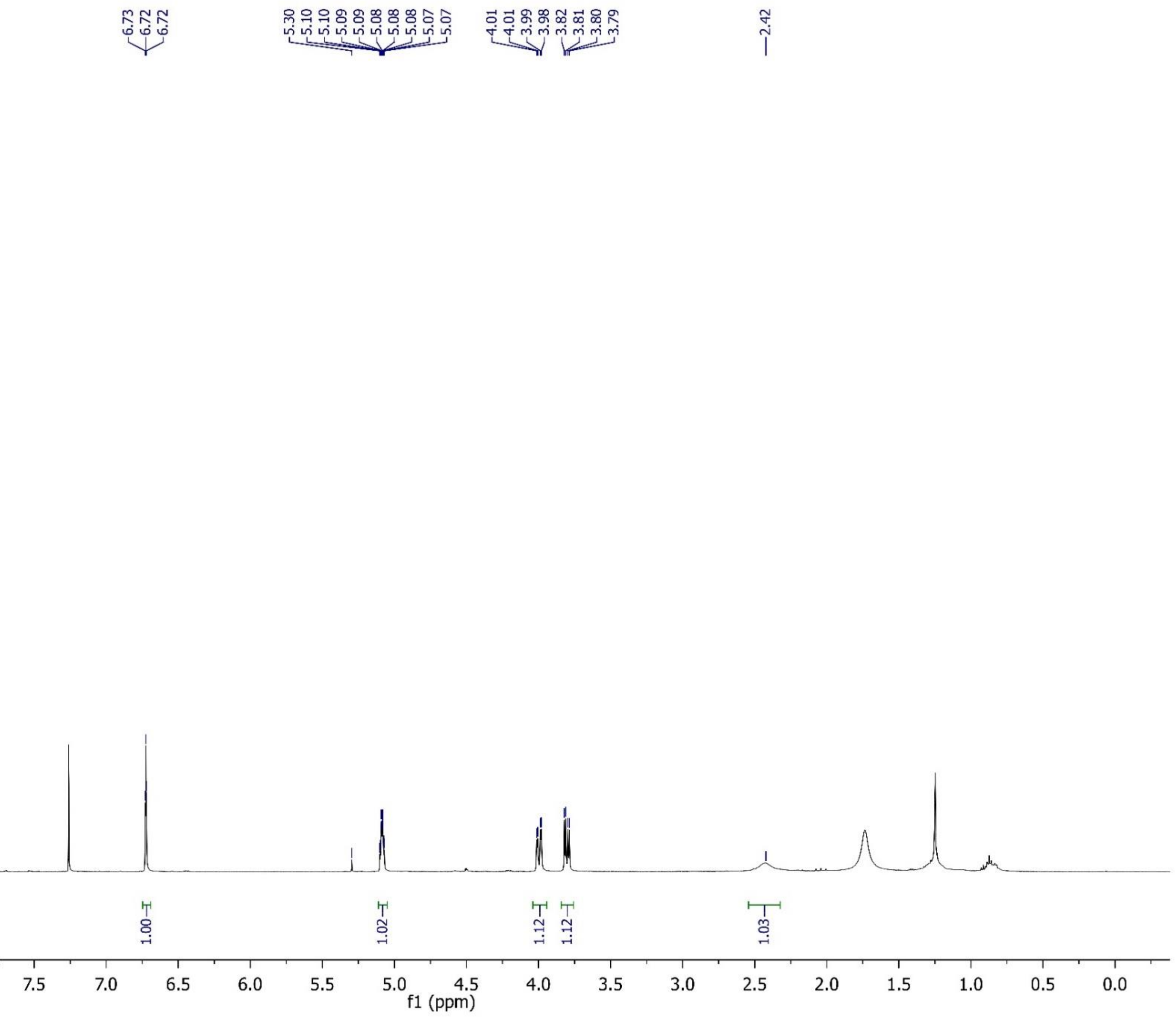

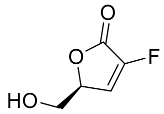

17

^{13}C NMR spectrum

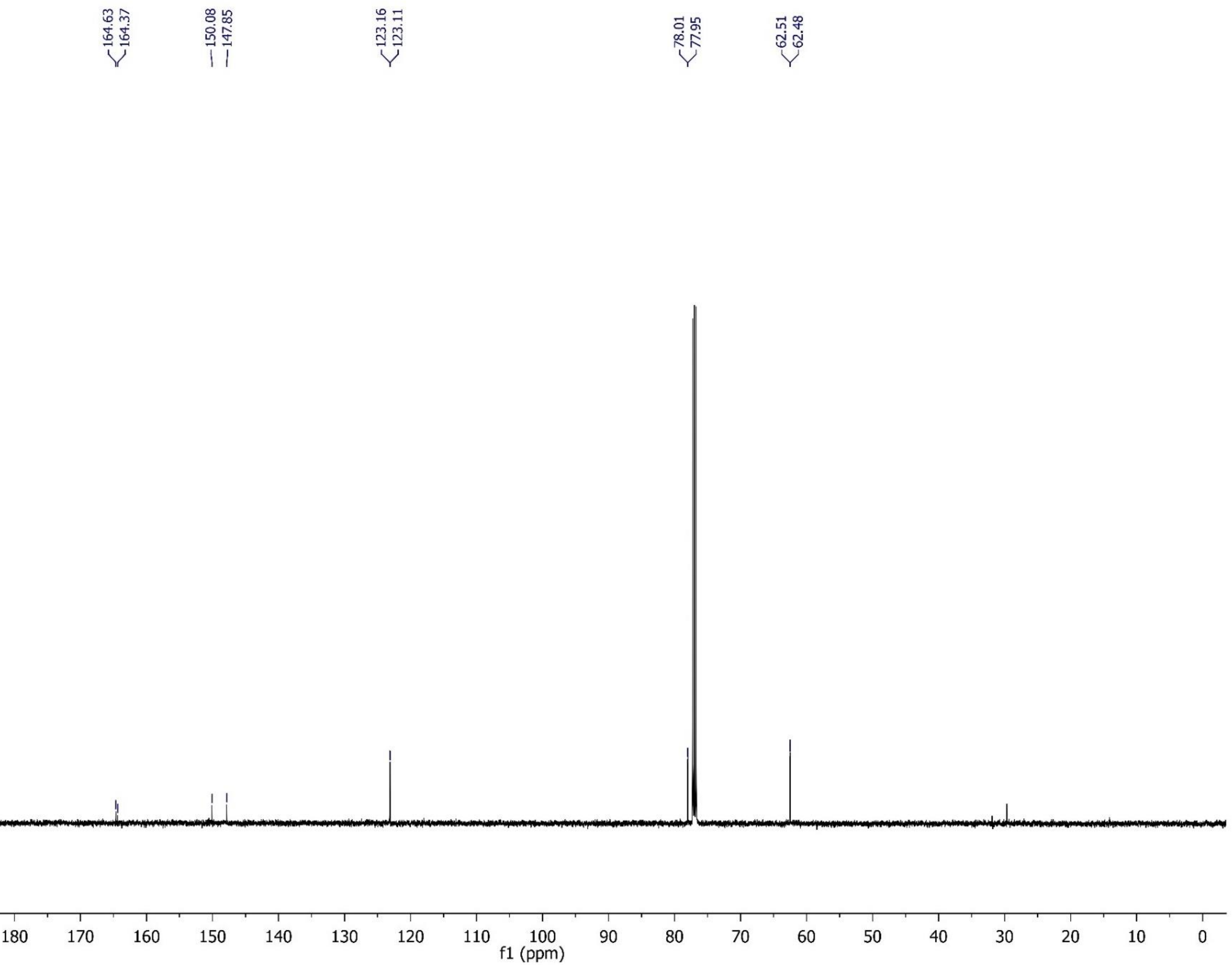

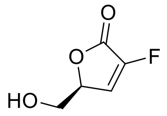

17

^{19}F NMR spectrum

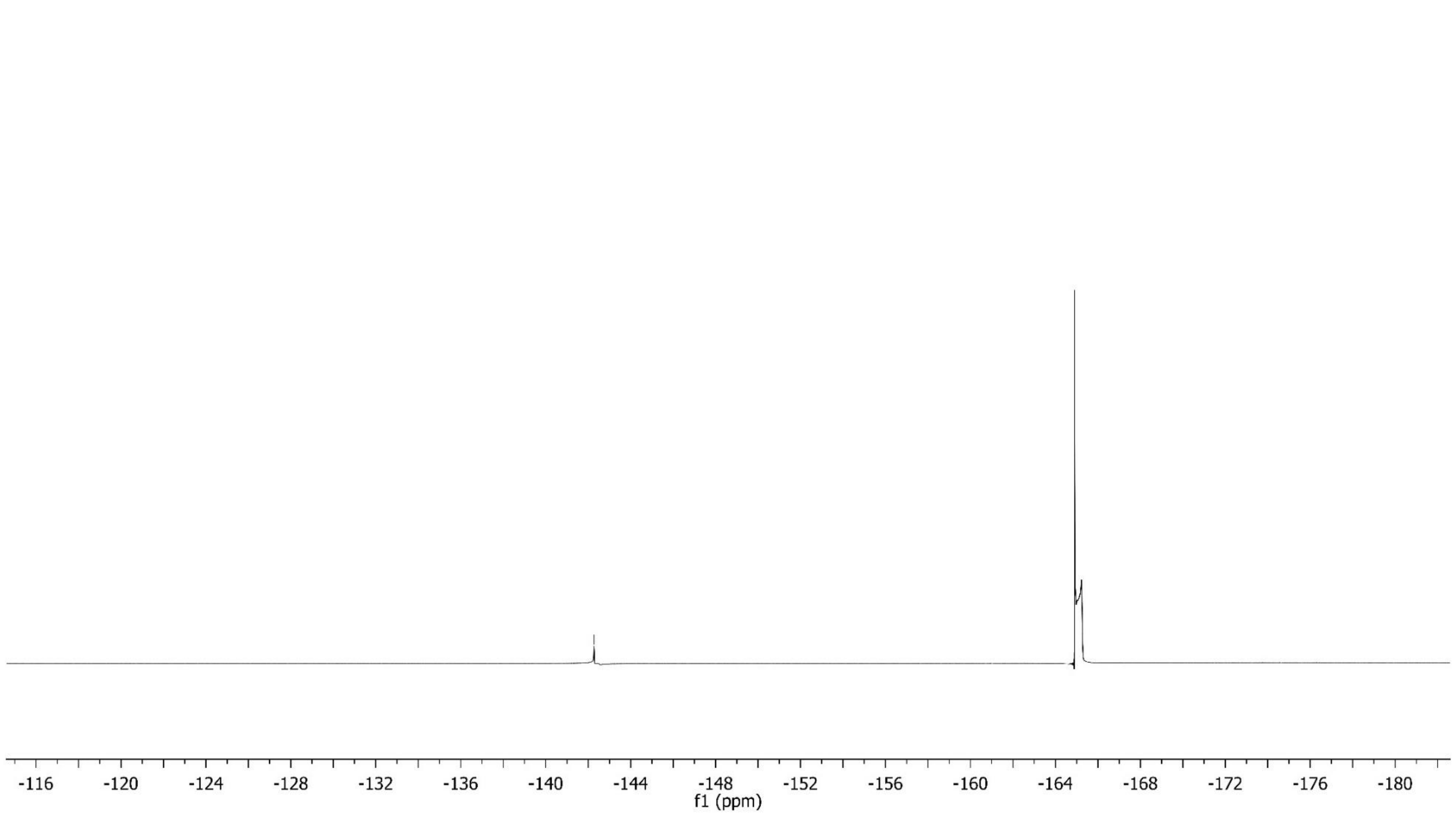

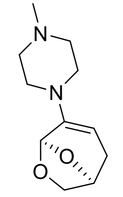

9b

^{1}H NMR spectrum

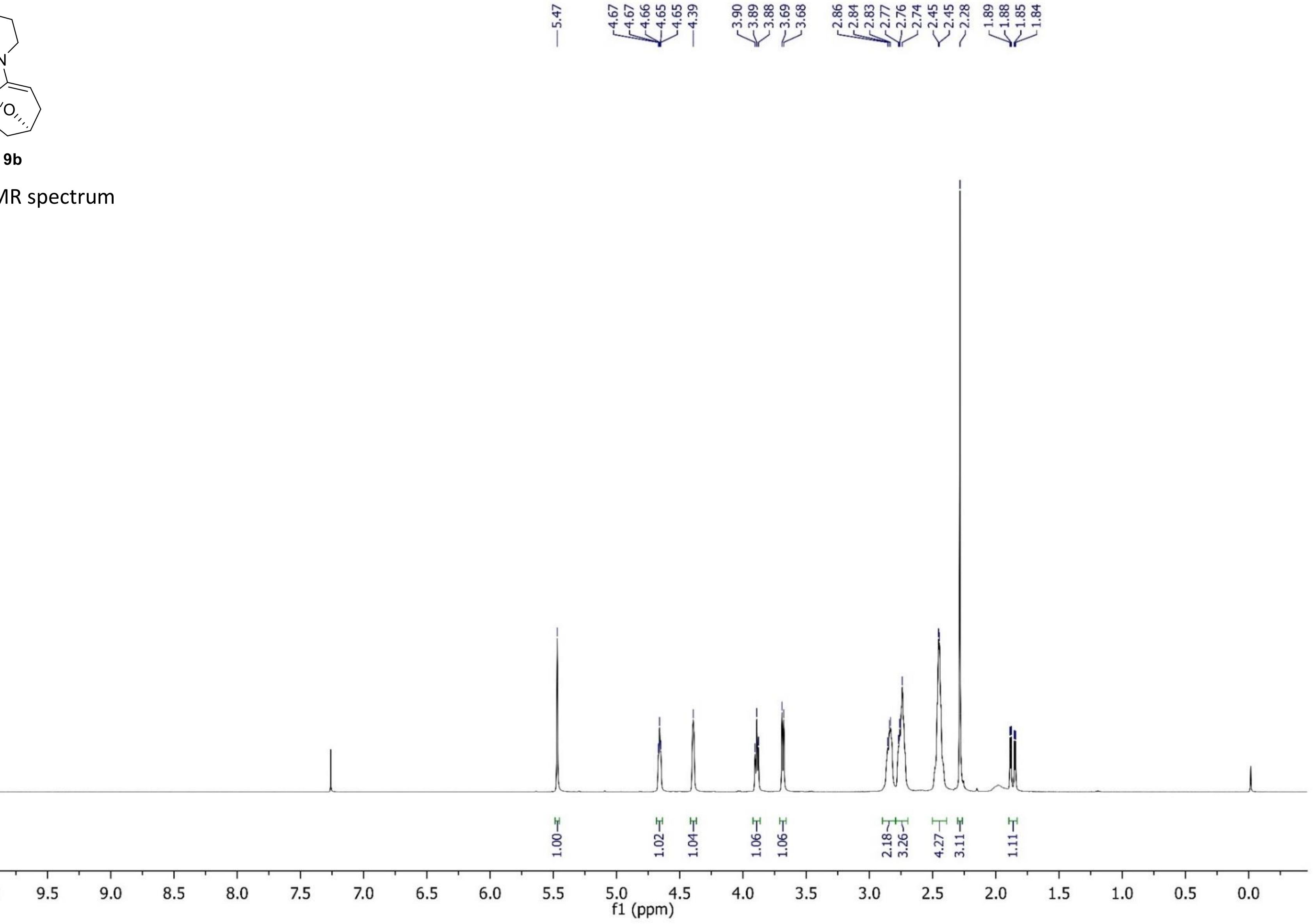

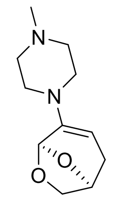

9b

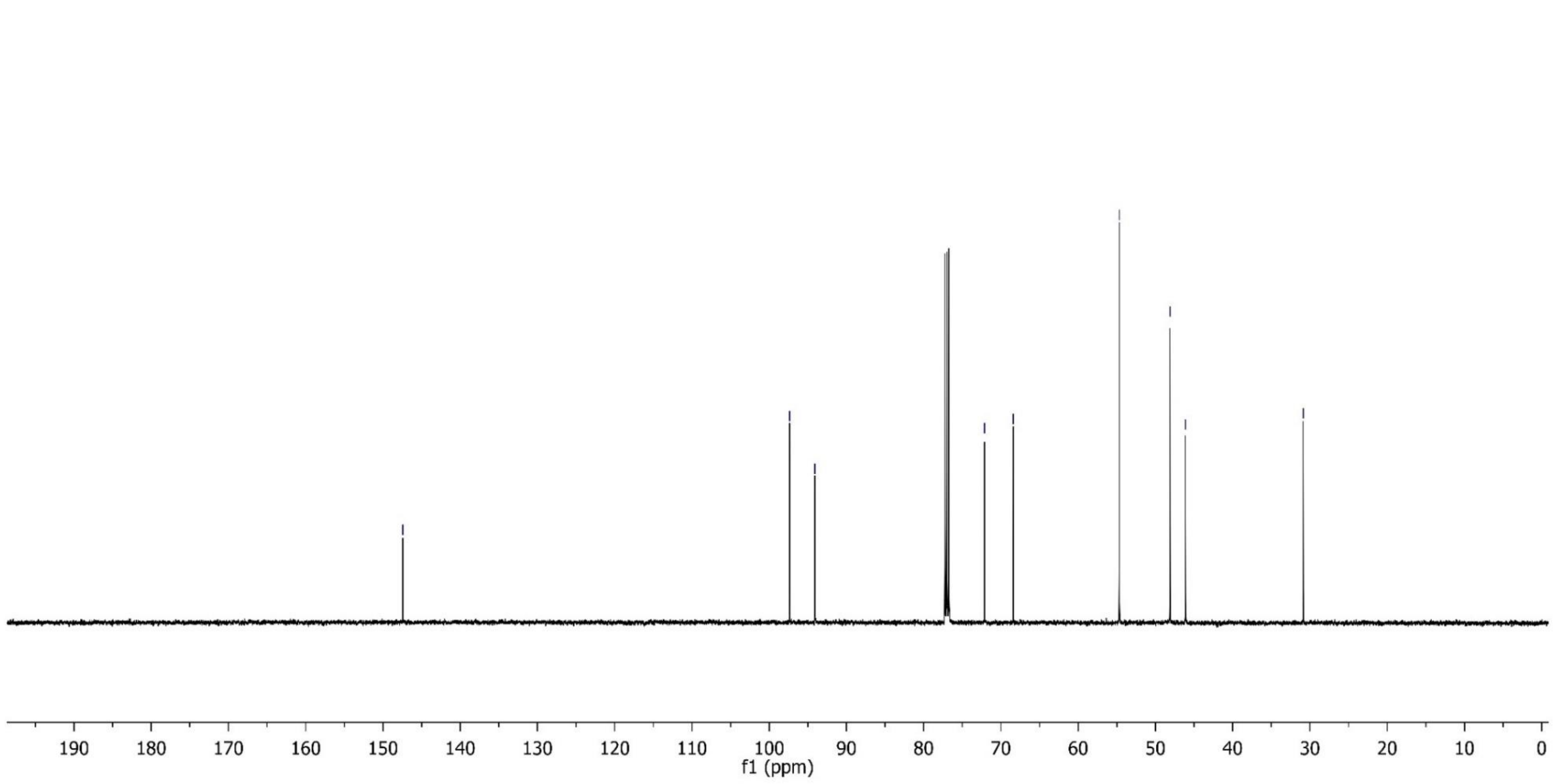

^{13}C NMR spectrum

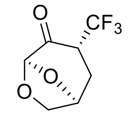

19

^{1}H NMR spectrum

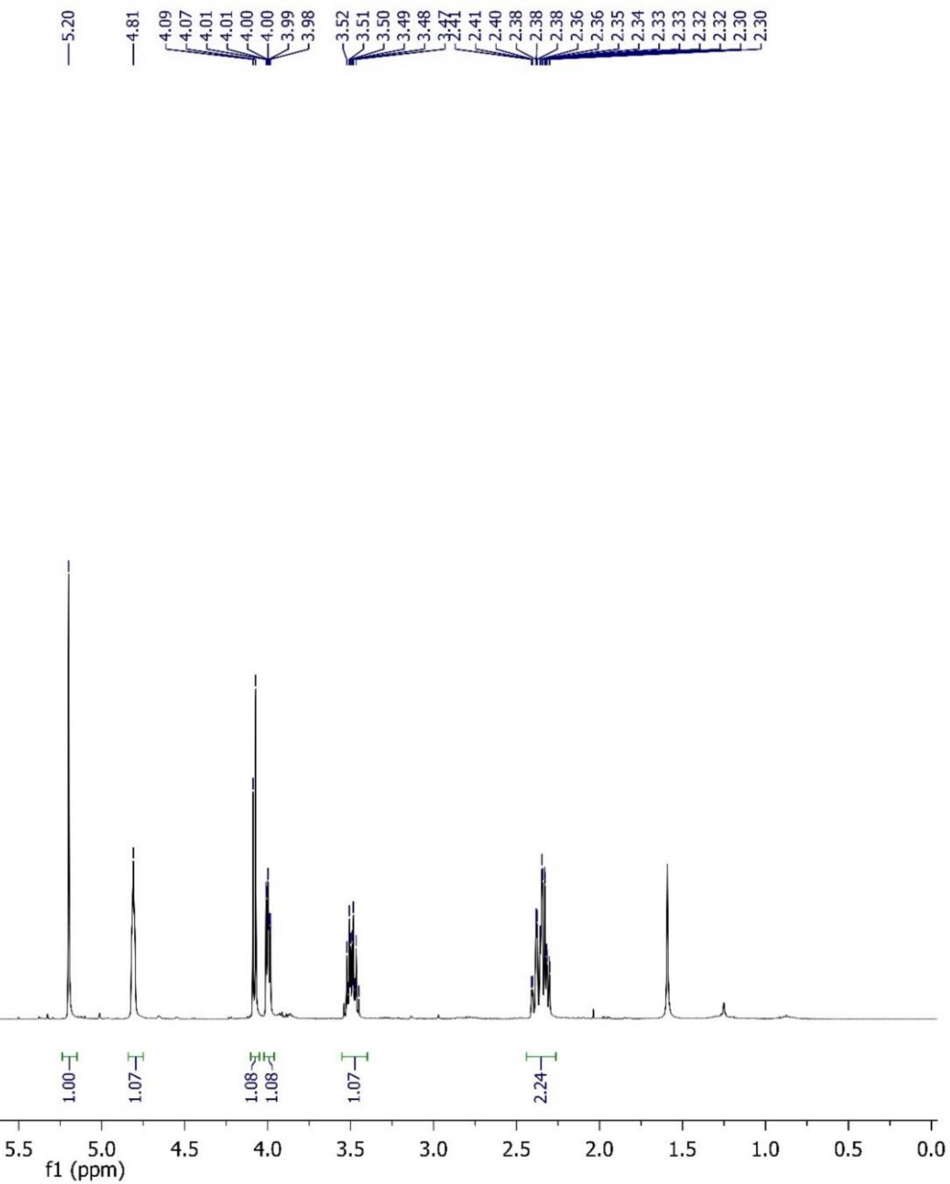

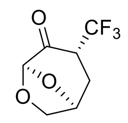

19

^{13}C NMR spectrum

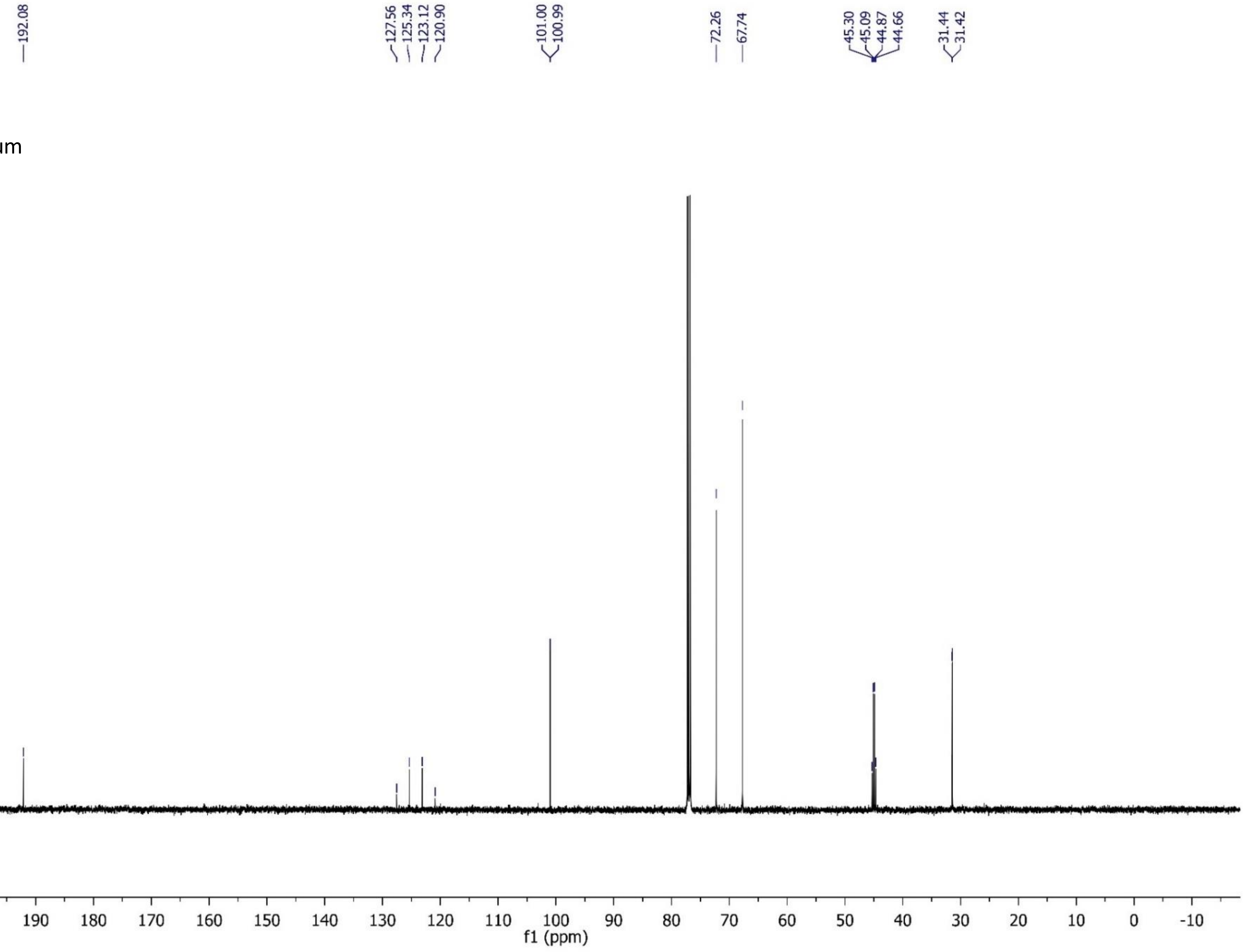

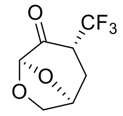

19

^{19}F NMR spectrum

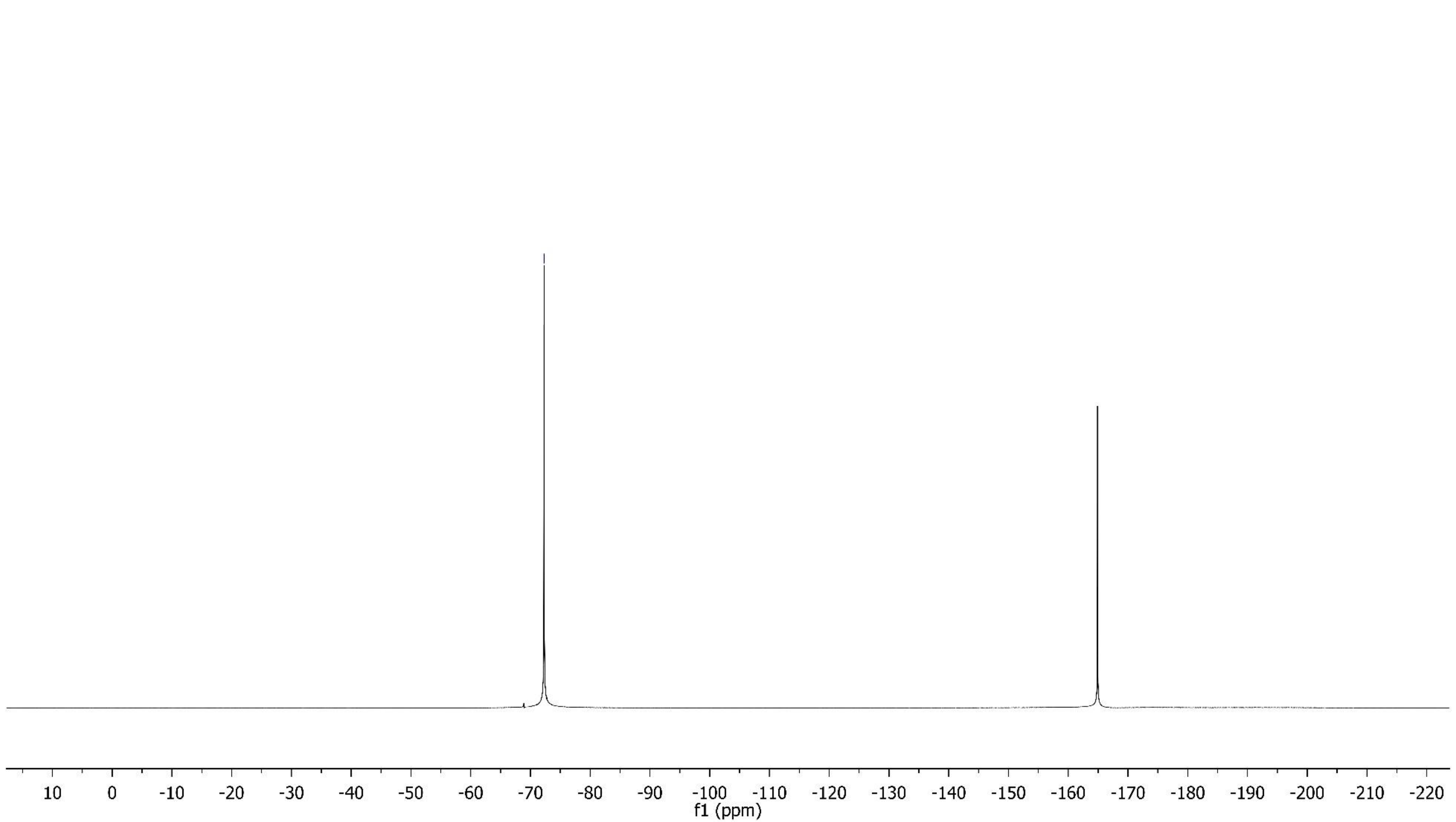

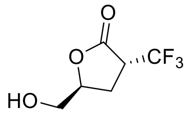

20

^{1}H NMR spectrum

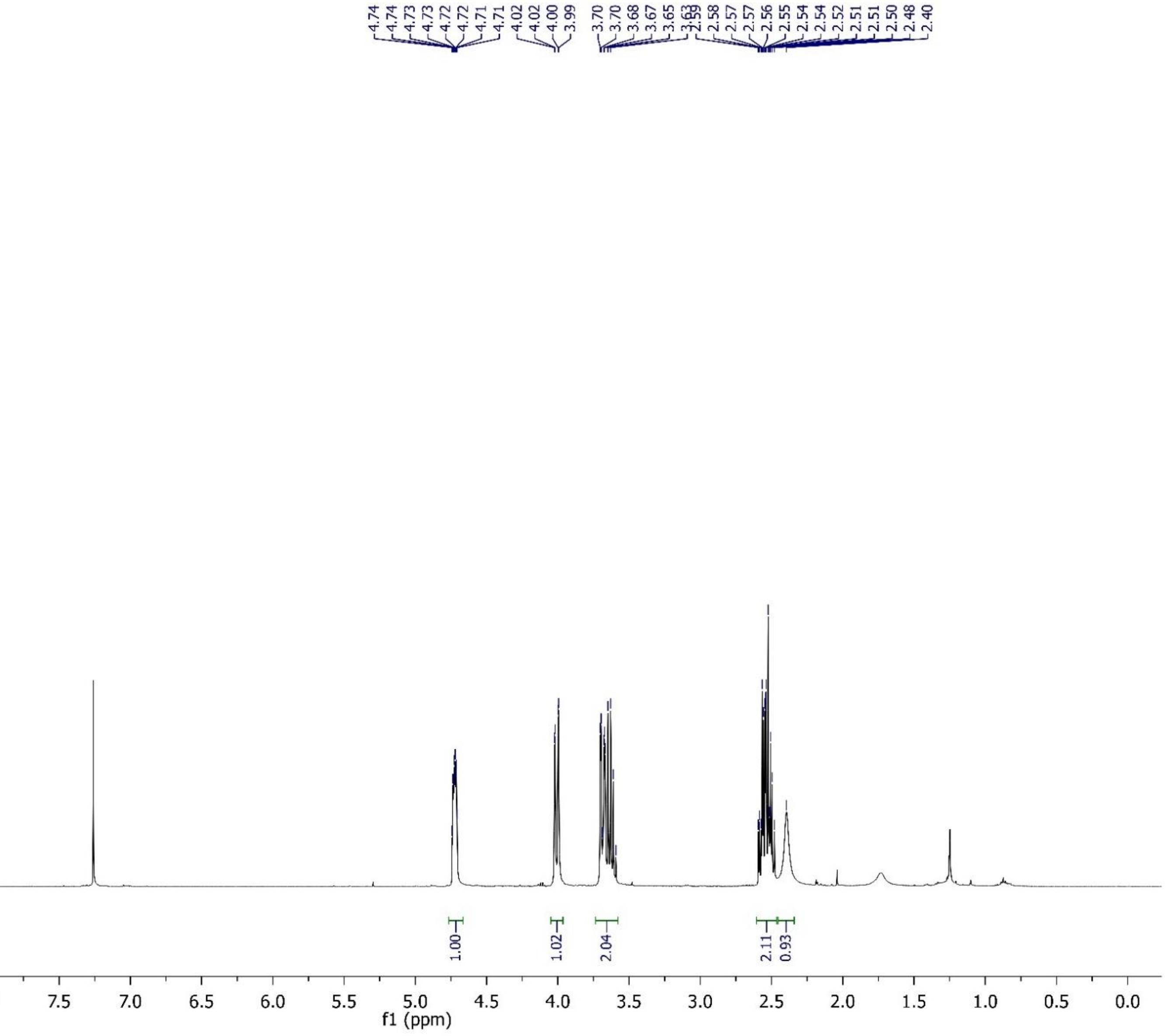

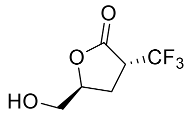

20

^{13}C NMR spectrum

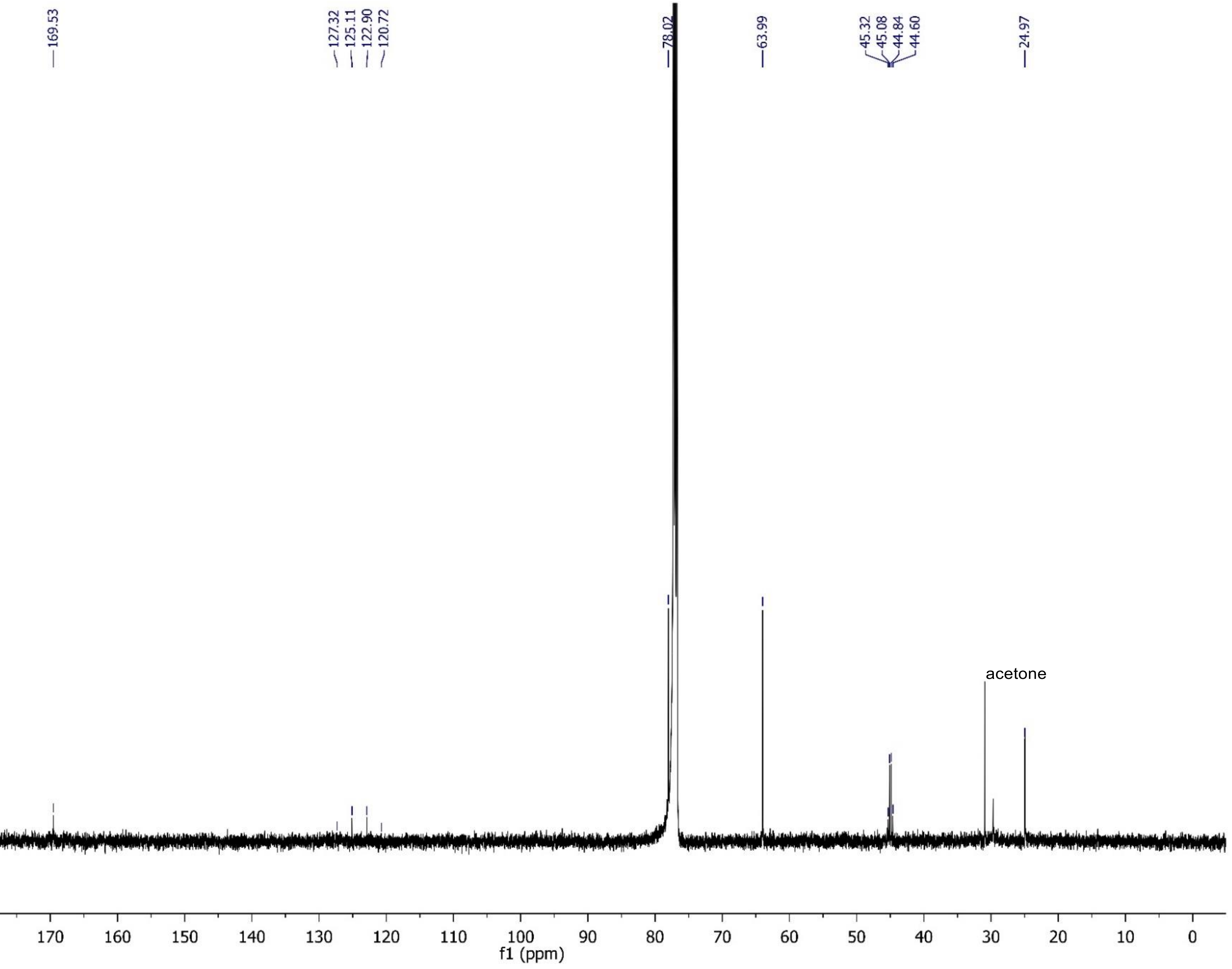

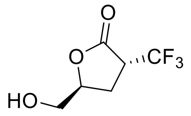

20

^{19}F NMR spectrum

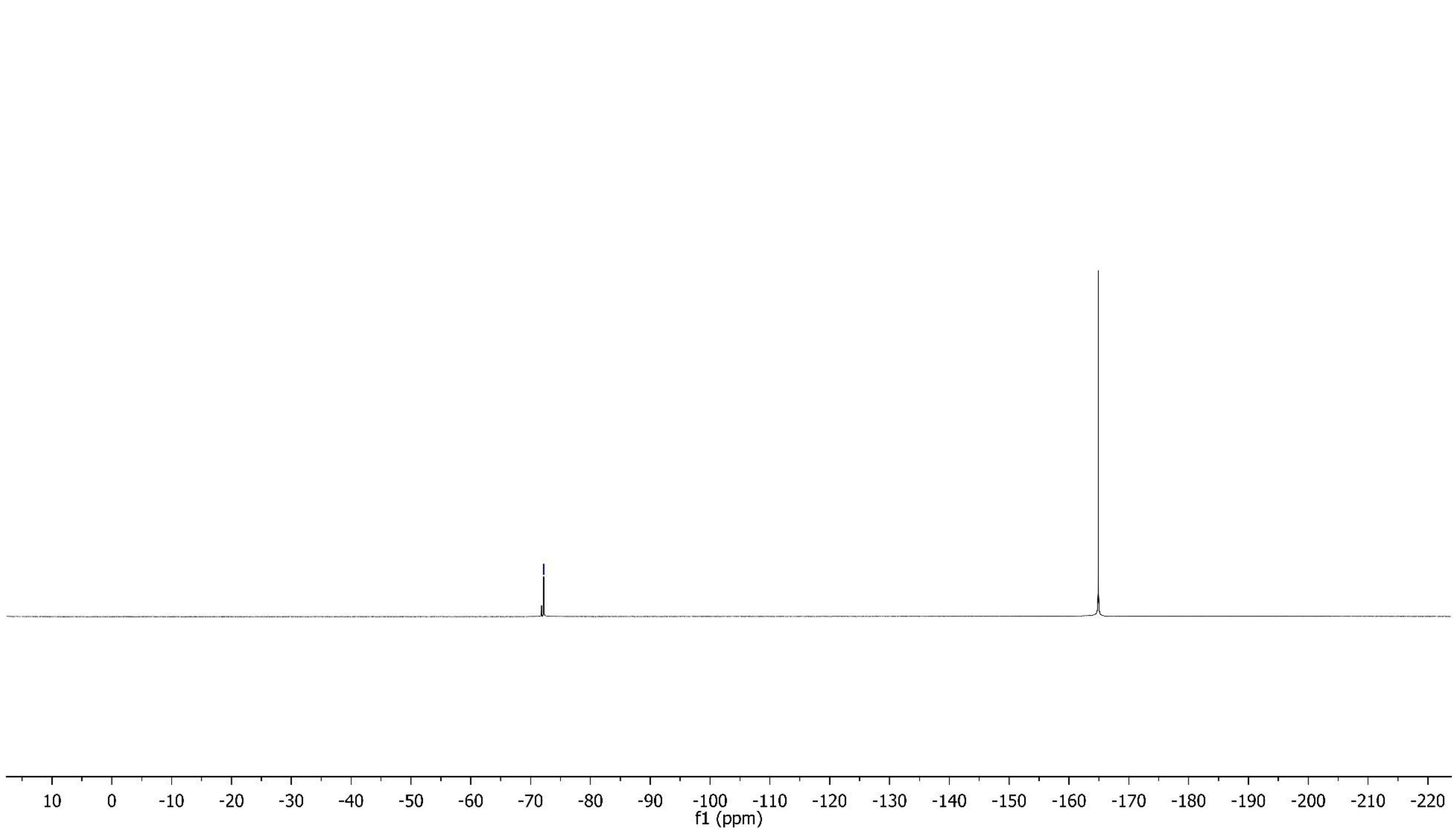

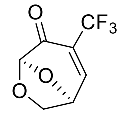

21

^1H NMR spectrum

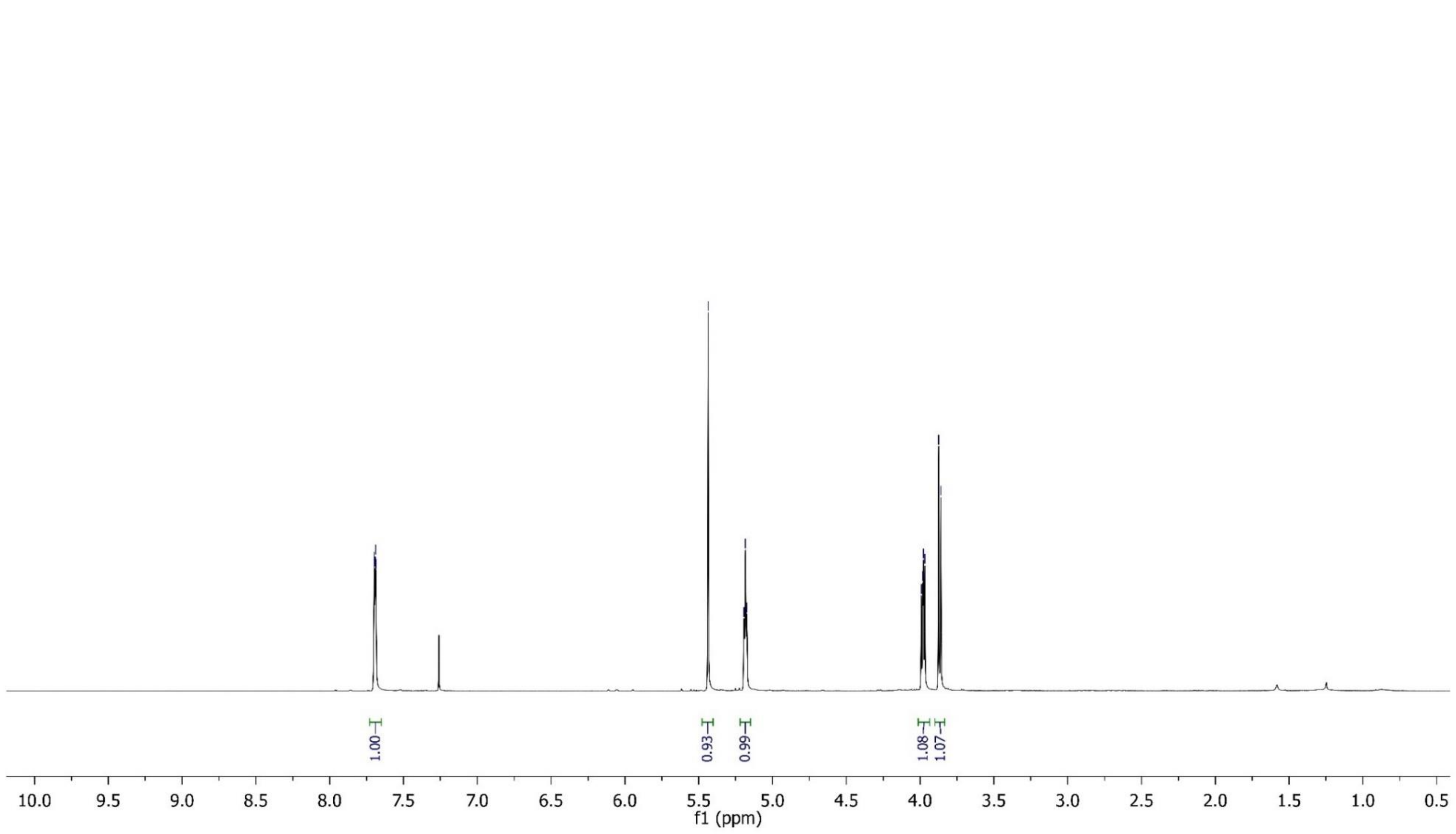

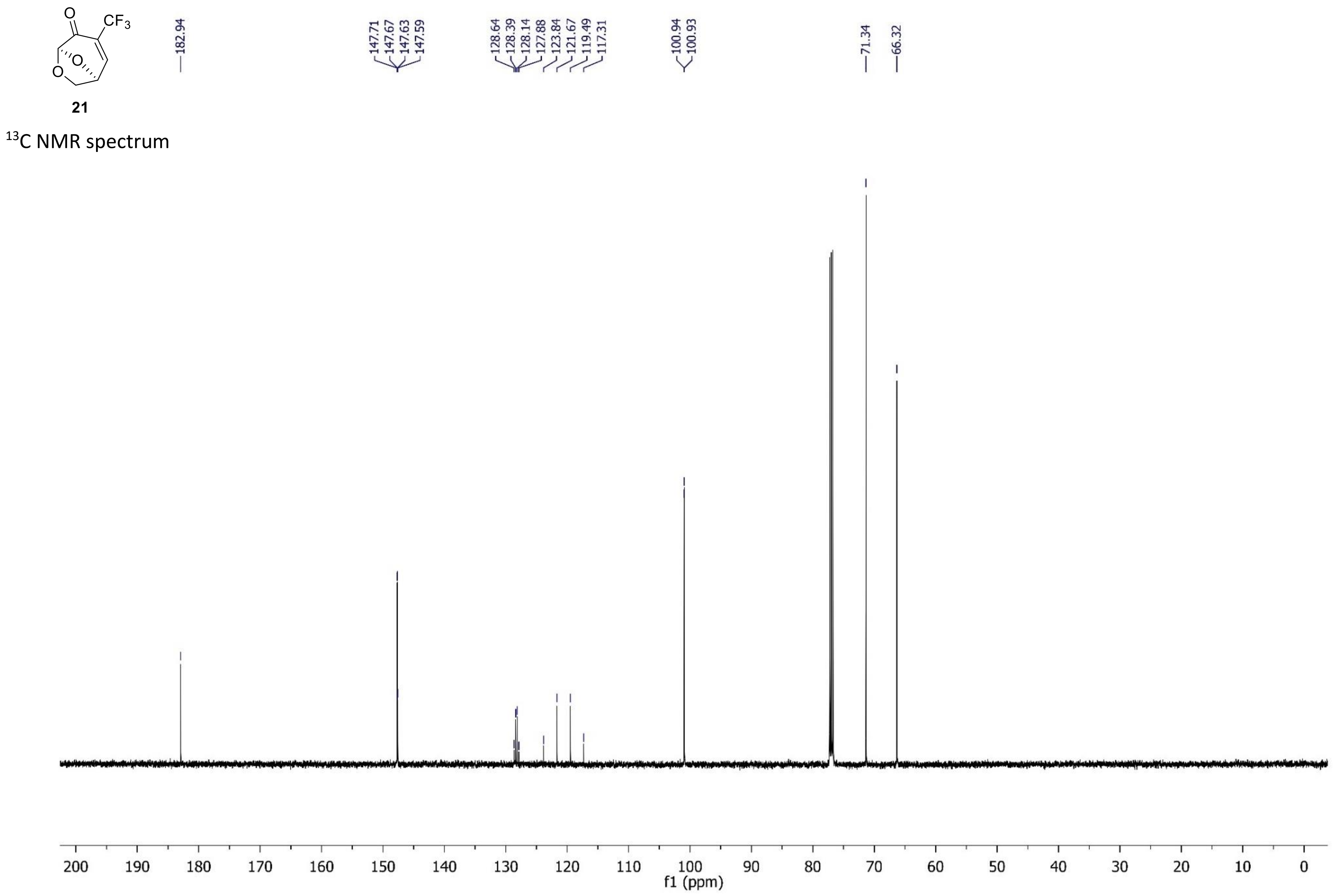

21

^{13}C NMR spectrum

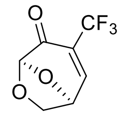

21

^{19}F NMR spectrum

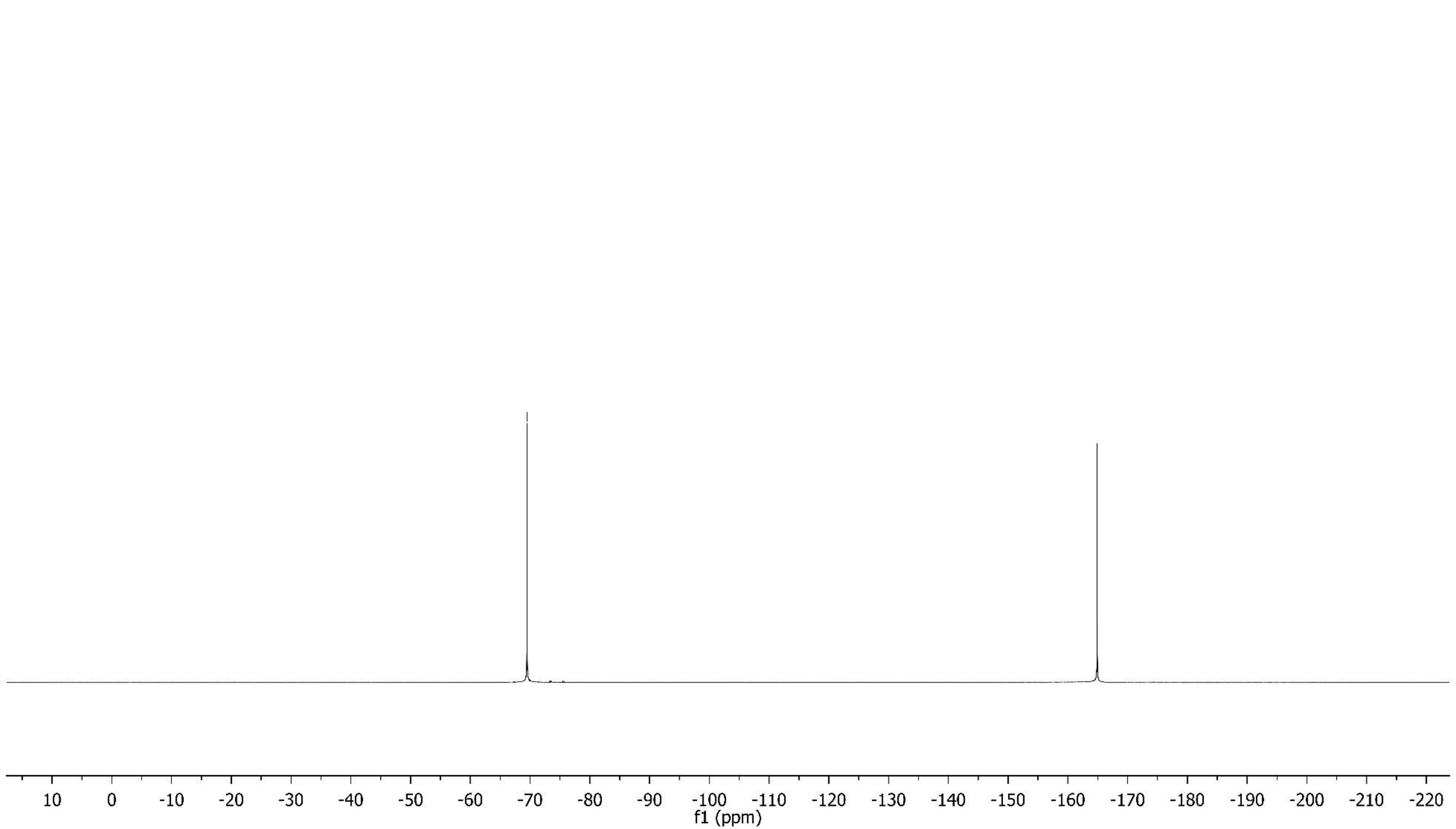

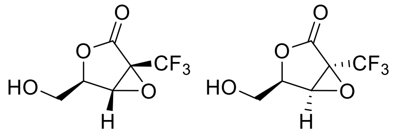

22

^{1}H NMR spectrum

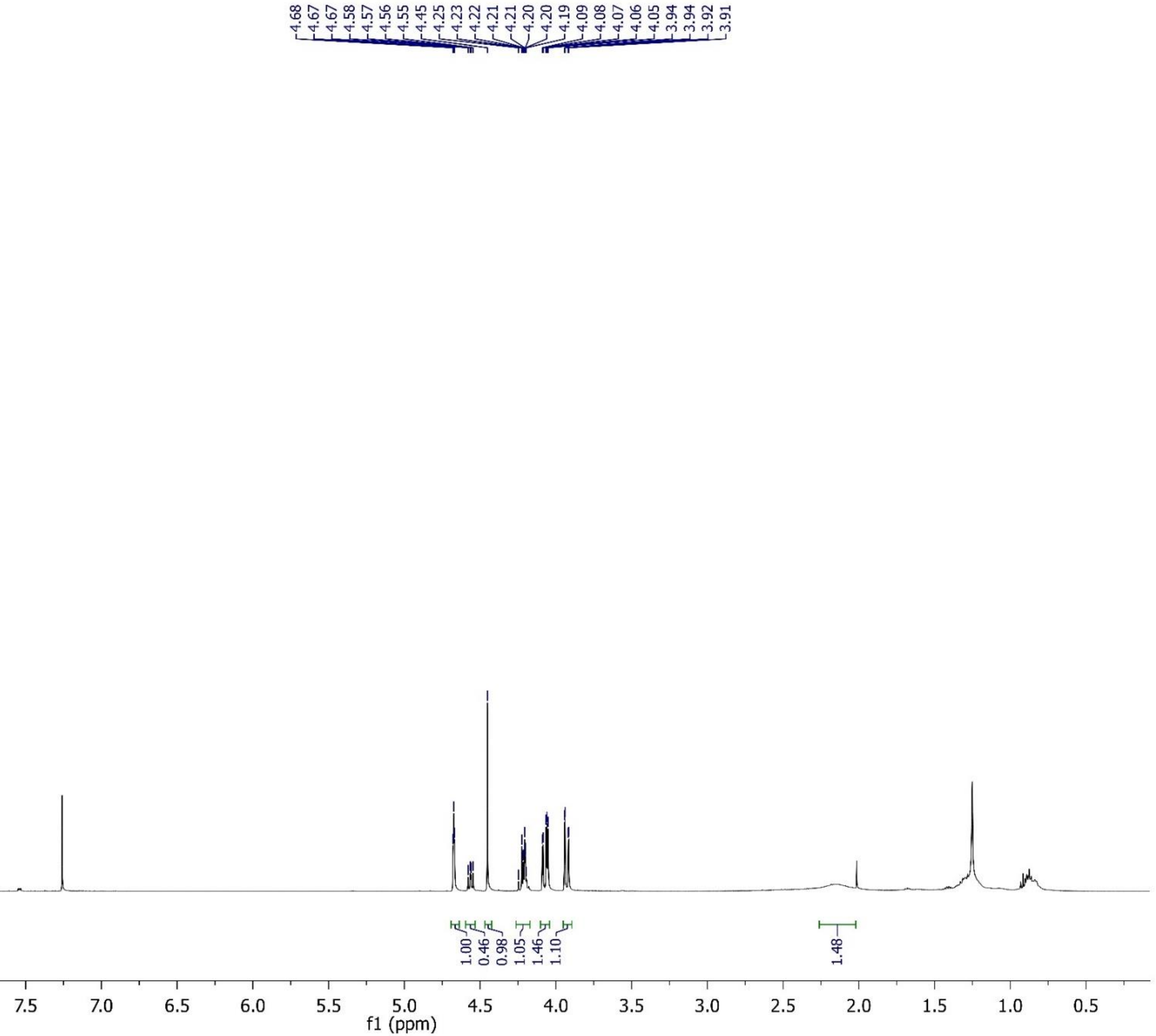

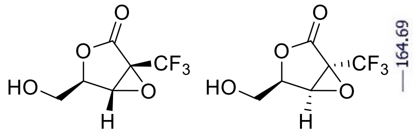

22

^{13}C NMR spectrum

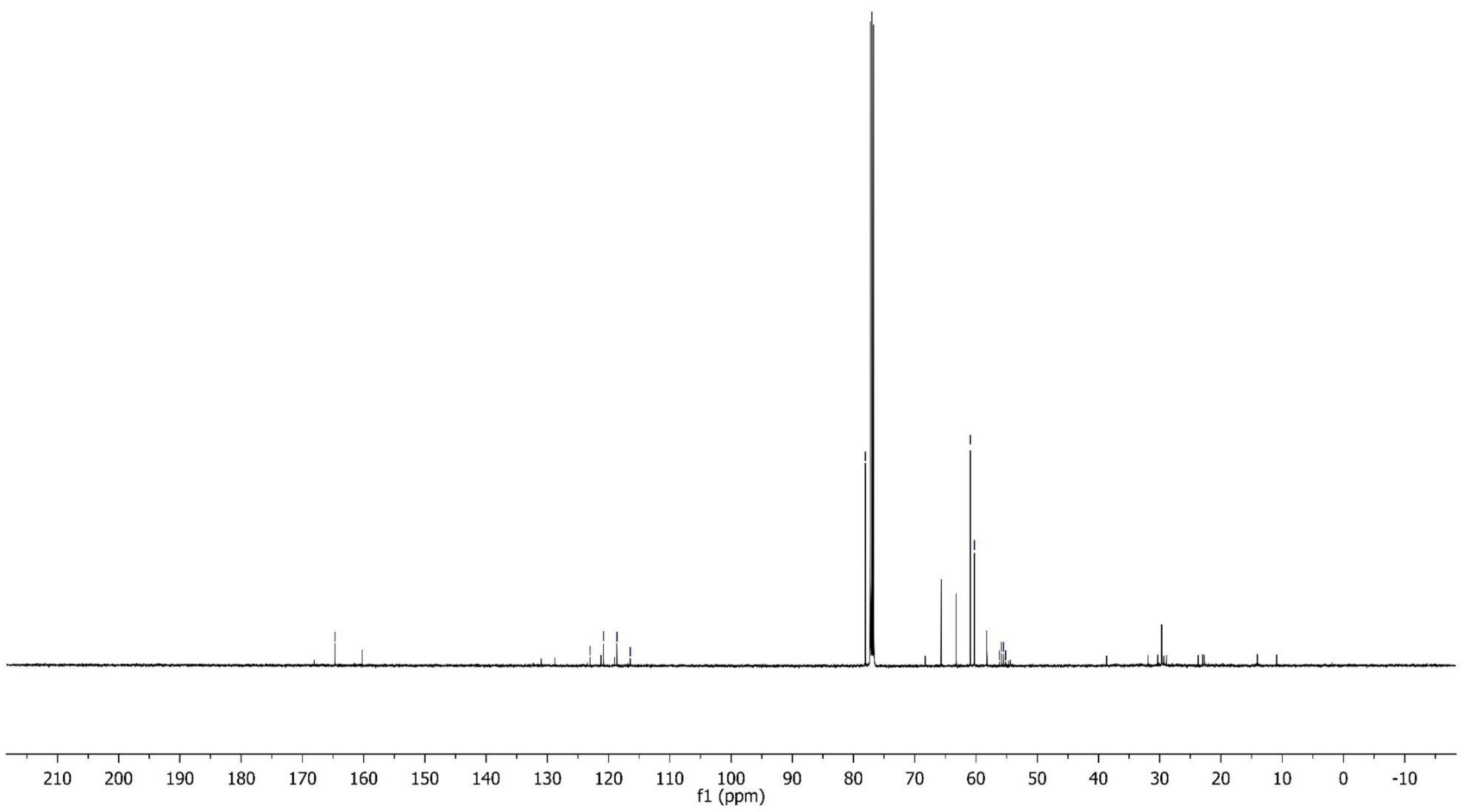

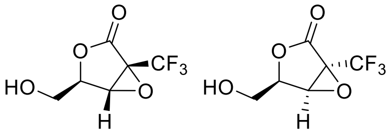

22

^{19}F NMR spectrum

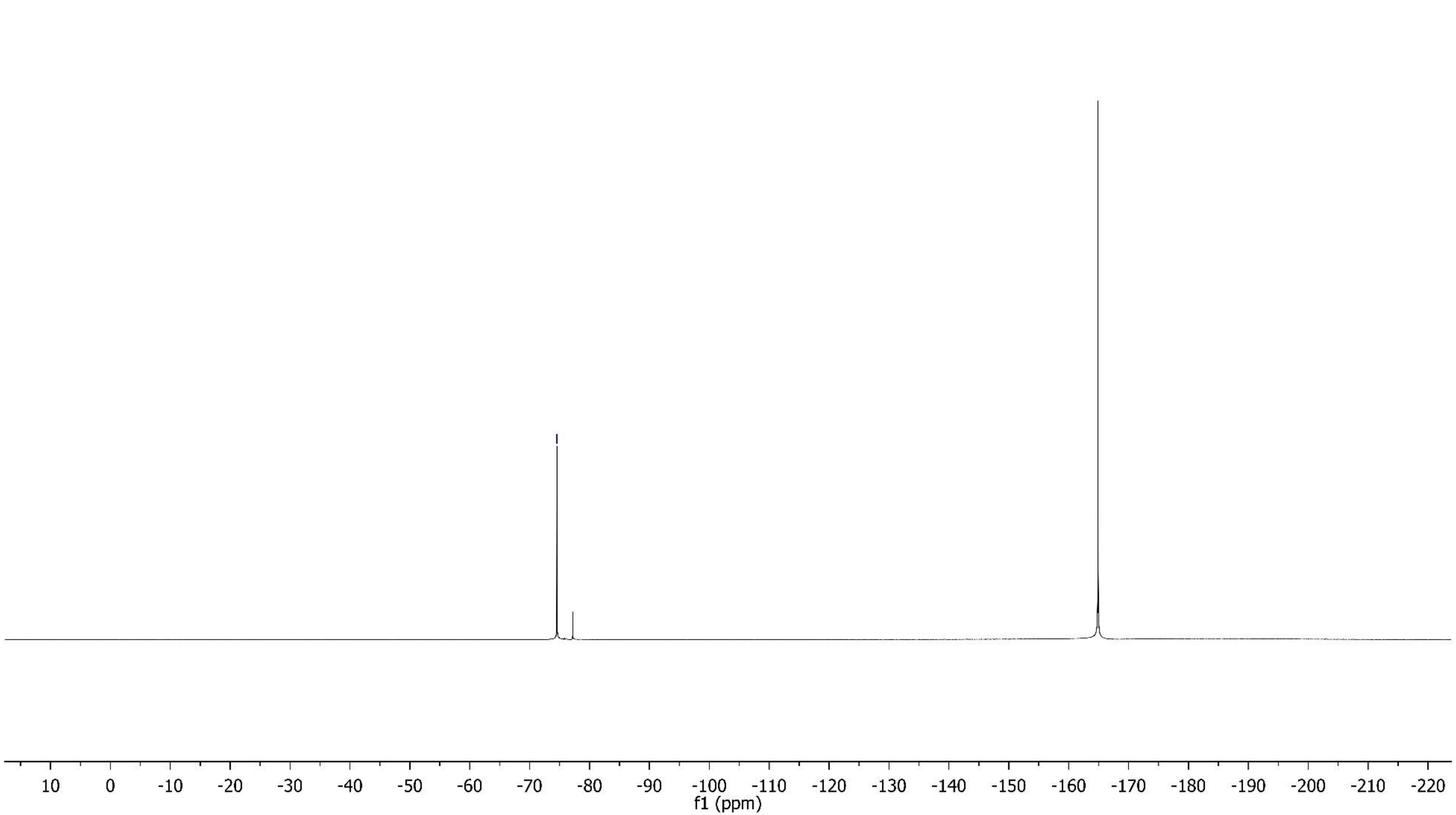

Supplement: File 1 — Experimental section, characterization data and copies of spectra. [file Beilstein_J_Org_Chem-21-2297-s001.pdf]
